# mTOR coordinates transcriptional programs and mitochondrial metabolism of activated T_reg_ subsets to protect tissue homeostasis

**DOI:** 10.1038/s41467-018-04392-5

**Published:** 2018-05-29

**Authors:** Nicole M. Chapman, Hu Zeng, Thanh-Long M. Nguyen, Yanyan Wang, Peter Vogel, Yogesh Dhungana, Xiaojing Liu, Geoffrey Neale, Jason W. Locasale, Hongbo Chi

**Affiliations:** 1 Department of Immunology, St. Jude Children’s Research Hospital, 262 Danny Thomas Place, MS 351, Memphis, TN 38105 USA; 2 Department of Pathology, St. Jude Children’s Research Hospital, 262 Danny Thomas Place, MS 250, Memphis, TN 38105 USA; 3 Department of Pharmacology & Cancer Biology, Duke University School of Medicine, Levine Science Research Center C266, Box 3813, Durham, NC 27710 USA; 4 Hartwell Center for Bioinformatics and Biotechnology, St. Jude Children’s Research Hospital, 262 Danny Thomas Place, MS 312, Memphis, TN 38105 USA

## Abstract

Regulatory T (T_reg_) cells derived from the thymus (tT_reg_) and periphery (pT_reg_) have central and distinct functions in immunosuppression, but mechanisms for the generation and activation of T_reg_ subsets in vivo are unclear. Here, we show that mechanistic target of rapamycin (mTOR) unexpectedly supports the homeostasis and functional activation of tT_reg_ and pT_reg_ cells. mTOR signaling is crucial for programming activated T_reg_-cell function to protect immune tolerance and tissue homeostasis. T_reg_-specific deletion of mTOR drives spontaneous effector T-cell activation and inflammation in barrier tissues and is associated with reduction in both thymic-derived effector T_reg_ (eT_reg_) and pT_reg_ cells. Mechanistically, mTOR functions downstream of antigenic signals to drive IRF4 expression and mitochondrial metabolism, and accordingly, deletion of mitochondrial transcription factor A (Tfam) severely impairs T_reg_-cell suppressive function and eT_reg_-cell generation. Collectively, our results show that mTOR coordinates transcriptional and metabolic programs in activated T_reg_ subsets to mediate tissue homeostasis.

## Abstract (web summary)

The authors previously showed that mTOR controls the function of regulatory T cells. Here they show how this mTOR signaling orchestrates homeostasis of T_reg_-cell subsets and prevents fatal autoimmunity.

## Introduction

Regulatory T (T_reg_) cells expressing the transcription factor Foxp3 suppress conventional T-cell responses to establish self-tolerance, prevent autoimmunity, and maintain tissue homeostasis^1,2^. Foxp3 deficiency eliminates T_reg_-cell development and function, leading to autoimmune diseases characterized by excessive T helper 1 (T_H_1), T_H_2, or T_H_17 responses, and germinal center (GC) B-cell reactions driven by T follicular helper (T_FH_) cells^3–5^. Thymic-derived T_reg_ (tT_reg_) cells exit the thymus and populate peripheral tissues, where resting T_reg_ cells [also called central T_reg_ (cT_reg_) cells] are activated in response to antigen and inflammatory cues^6–9^. These activation signals increase effector molecule expression and induce transcription factors that define the selective suppressive functions and tissue localization of activated T_reg_ cells [also known as effector T_reg_ (eT_reg_) cells]^5,10–15^. Peripherally-derived T_reg_ (pT_reg_) cells are a developmentally distinct population of activated T_reg_ cells that arises from the naive CD4^+^ T-cell pool and inhibit T_H_2 or T_H_17 responses at mucosal sites^6,16–19^. The transcription factor interferon regulatory factor 4 (IRF4) is expressed in both eT_reg_ and pT_reg_ cells in vivo and is an essential positive regulator of their homeostasis and function^7,15,17,20–22^. IRF4 expression and function are induced by TCR signals in T_reg_ cells by incompletely understood mechanisms^7,8,22^.

Metabolic rewiring is important for T-cell fate decisions, but the metabolic programs regulating T_reg_-cell activation and specialization remain uncertain^23^. The activation of the mechanistic target of rapamycin (mTOR) induces metabolic reprogramming necessary for conventional T-cell activation and differentiation^23,24^. In contrast, mTOR appears to antagonize T_reg_-cell differentiation and expansion in vitro and suppressive activity in vivo^23,25,26^. Mechanistically, inhibition of mTOR upregulates fatty acid oxidation, which supports mitochondrial respiration important for T_reg_-cell differentiation, proliferation, and survival in vitro^27,28^. Moreover, low levels of mTOR activation are needed to prevent excessive glycolysis that can impair T_reg_-cell survival and lineage stability^23^. Although the prevailing model is that mTOR activation hinders T_reg_-cell function, T_reg_ cells have higher basal levels of mTORC1 activation than conventional T cells^29,30^, which is essential for T_reg_-cell function in vivo^30^. Thus, mTOR-dependent metabolic programming might have context-dependent roles in different T_reg_-subsets or under distinct physiological conditions.

Here, we show that mTOR orchestrates activation-induced transcriptional and metabolic signatures that are essential for T_reg_-cell activation and function. We find that either acute or chronic inhibition of mTOR disrupts T_reg_-cell suppressive activity and leads to uncontrolled conventional T-cell activation. In line with this observation, mucosal CD4^+^ T-cell responses, including T_H_2 responses, are increased when T_reg_ cells lose mTOR, associated with a loss of eT_reg_ and pT_reg_ cells in mucosal sites. Mechanistically, mTOR mediates T_reg_-cell activation and suppressive activity by promoting IRF4 expression and mitochondrial metabolism. Indeed, disruption of mitochondrial metabolism severely impairs the suppressive function of activated T_reg_ cells and their homeostasis in tissues. Collectively, our results show that mTOR controls peripheral tolerance by integrating transcriptional and metabolic programs critical for the homeostasis and suppressive activity of activated T_reg_ cells.

## Results

### mTOR promotes activated T_reg_-cell suppressive activity

**Fig. 1:**
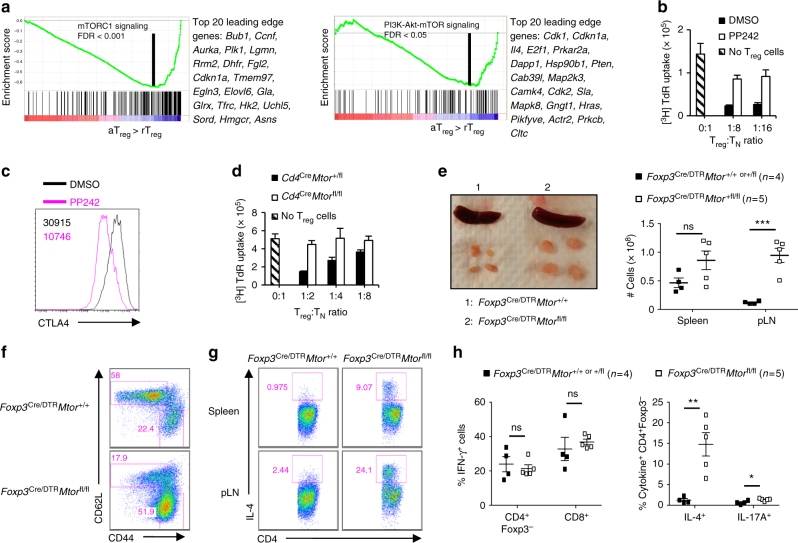
mTOR is essential for activated T_reg_ cell function. **a** Enrichment plots of the Hallmark mTORC1 (left) and Hallmark PI3K-Akt-mTOR (right) signaling pathways in activated T_reg_ (aT_reg_) compared to resting T_reg_ (rT_reg_) cells, identified by gene set enrichment analysis (GSEA). The top 20 enriched genes in each pathway (position indicated by the vertical black line) are listed to the right of each plot. **b** In vitro suppressive activity of T_reg_ cells activated in the presence or absence of PP242. T_N_: naive CD4^+^ T cells. **c** Flow cytometry analysis of CTLA4 expression in T_reg_ cells activated in the presence or absence of PP242. **d** In vitro suppressive activity of T_reg_ cells isolated from *Cd4*^Cre^*Mtor*^+/+ or +/fl^ or *Cd4*^Cre^*Mtor*^fl/fl^ mice. **e** Representative image of lymphadenopathy in *Foxp3*^Cre/DTR^*Mtor*^fl/fl^ mice after DT treatment (left). Right, cell numbers of the spleen and peripheral lymph nodes (pLN) of *Foxp3*^Cre/DTR^*Mtor*^+/+ or +/fl^ or *Foxp3*^Cre/DTR^*Mtor*^fl/fl^ mice. **f** Flow cytometry analysis of naive and effector/memory CD4^+^Foxp3-YFP^−^ in pLN. **g**, **h** Cells from the spleen and pLN of *Foxp3*^Cre/DTR^*Mtor*^+/+ or +/fl^ or *Foxp3*^Cre/DTR^*Mtor*^fl/fl^ mice that received DT treatments were stimulated using PMA and ionomycin for 4–5 h. **g** Flow cytometry analysis of IL-4-producing CD4^+^ T cells in the spleen and pLN. **h** Quantification of IFN-γ^+^ CD4^+^Foxp3^−^ and CD8^+^ T cells or IL-4^+^ and IL-17A^+^ CD4^+^Foxp3^−^ T cells in the spleen. Error bars show mean ± s.e.m. *P < 0.05; **P < 0.01; ***P < 0.001; ns, not significant; unpaired, two-tailed Student’s *t*-test. Data are representative of three (**c**) or four (**e**–**g**) biological replicates from three (**c**) or two (**e**−**g**) independent experiments. Data are quantified from three technical replicates representative of three independent experiments (**b**, **d**) or four or five biological replicates per group as indicated, compiled from two independent experiments (**e**, **h**). Numbers indicate percentage of cells in gates

T_reg_ cells activated in vivo have enhanced suppressive activity critical for immune homeostasis^7,8,31,32^, yet the molecular events controlling T_reg_-cell activation remain to be fully defined. To identify pathways associated with increased suppressive function of T_reg_ cells, we mined a published dataset of activated T_reg_ cells isolated from diphtheria toxin (DT)-treated *Foxp3*^DTR^ mice (DTR, diphtheria toxin receptor)^32^. Gene set enrichment analysis (GSEA) revealed that the hallmark mTORC1 and PI3K-Akt-mTOR signaling pathways were among the most significantly (false discovery rate, FDR < 0.05) upregulated gene sets in activated vs. resting T_reg_ cells (Fig. 1a). Thus, increased T_reg_-cell suppressive activity is correlated with enhanced mTOR signaling. To rigorously test the function of mTOR for the suppressive activity of activated T_reg_ cells, we activated T_reg_ cells in vitro in the presence or absence of the mTOR inhibitor, PP242. We found that acute inhibition of mTOR diminished the ability of activated T_reg_ cells to suppress conventional T-cell proliferation (Fig. 1b) and to express the immunosuppressive molecule CTLA4 (Fig. 1c), indicating a kinase-dependent function of mTOR in T_reg_-cell function. Accordingly, the suppressive activity of T_reg_ cells isolated from *Cd4*^Cre^*Mtor*^fl/fl^ mice was dampened (Fig. 1d). Thus, mTOR is essential for the suppressive function of T_reg_ cells in vitro.

To establish a role for mTOR in T_reg_-cell function in vivo, we generated female *Foxp3*^Cre/DTR^*Mtor*^fl/fl^ mosaic mice. These mice express a floxed *Mtor* allele^24^, whose expression can be deleted by Cre recombinase driven under the *Foxp3* promoter (denoted as *Foxp3*^Cre^)^33^, resulting in the deletion of mTOR within T_reg_ cells after they have expressed Foxp3. Acute depletion of DTR-expressing T_reg_ cells with DT forces the remaining *Foxp3*^Cre^-expressing T_reg_ cells to become activated, expand, and control immune homeostasis in adult mice^34^. Upon DT treatment, *Foxp3*^Cre/DTR^*Mtor*^fl/fl^ mosaic mice, but not their respective *Foxp3*^Cre/DTR^*Mtor*^+/+ or +/fl^ controls, developed inflammation associated with increased organ size and cell number, especially peripheral lymph nodes (Fig. 1e). Further, there were increased frequencies of CD44^hi^CD62L^lo^ effector/memory CD4^+^ in the peripheral lymph nodes (Fig. 1f). After DT treatment, *Foxp3*^Cre/DTR^*Mtor*^fl/fl^ mice had a profound enrichment for IL-4-producing and small but significant increase in IL-17A-producing, but not IFN-γ-producing, CD4^+^ T cells in the spleen (Fig. 1g, h). Similar observations were found in peripheral lymph nodes (Fig. 1g). Therefore, mTOR signaling is essential for the suppressive function of activated T_reg_ cells, and its acute deletion in T_reg_ cells leads to loss of immune homeostasis and the activation of T_H_2, and to a lesser extent, T_H_17 cells.

### T_reg_ cells require mTOR to prevent spontaneous autoimmunity

**Fig. 2:**
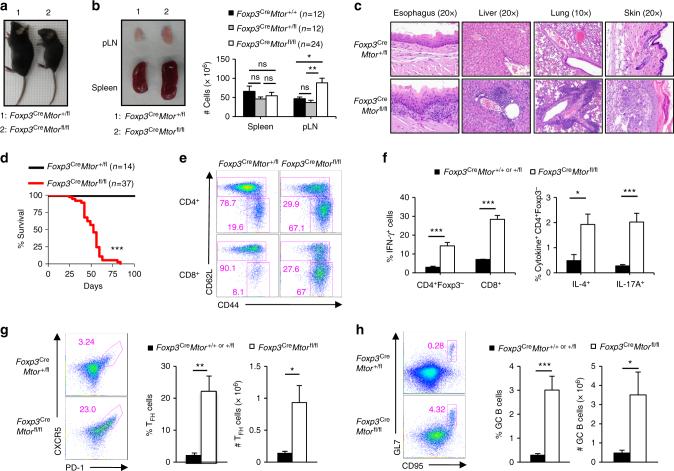
Disruption of mTOR in T_reg_ cells results in fatal autoimmunity. **a** Representative image of 47-day-old *Foxp3*^Cre^*Mtor*^+/fl^ and *Foxp3*^Cre^*Mtor*^fl/fl^ littermates. **b** Representative image of lymphadenopathy in 47-day-old *Foxp3*^Cre^*Mtor*^fl/fl^ mice (left). Right, cell numbers of the spleen and peripheral lymph nodes (pLN) of *Foxp3*^Cre^*Mtor*^+/+^, *Foxp3*^Cre^*Mtor*^+/fl^, or *Foxp3*^Cre^*Mtor*^fl/fl^ mice. The numbers of mice per group are indicated. **c** Representative hematoxylin and eosin staining of the indicated tissues from 6-week-old *Foxp3*^Cre^*Mtor*^+/fl^ and *Foxp3*^Cre^*Mtor*^fl/fl^ mice. The magnifications are indicated above the respective images for each tissue. **d** Survival curve of *Foxp3*^Cre^*Mtor*^+/fl^ and *Foxp3*^Cre^*Mtor*^fl/fl^ mice. The numbers of mice per group are indicated. **e** Flow cytometry analysis of naive and effector/memory CD4^+^Foxp3-YFP^–^ (depicted as CD4^+^) or CD8^+^ T-cell populations. **f** Splenocytes from *Foxp3*^Cre^*Mtor*^+/+ or +/fl^ and *Foxp3*^Cre^*Mtor*^fl/fl^ mice were stimulated using PMA and ionomycin for 4–5 h. Cytokine production by CD4^+^ and CD8^+^ T cells was assessed by flow cytometry and quantified. **g** Flow cytometry analysis of PD-1^+^CXCR5^+^ T_FH_ cells. Right, frequency and number of T_FH_ cells in *Foxp3*^Cre^*Mtor*^+/+ or +/fl^ and *Foxp3*^Cre^*Mtor*^fl/fl^ mice. **h** Flow cytometry analysis of CD95^+^GL7^+^ GC B cells. Right, frequency and number of GC B cells in *Foxp3*^Cre^*Mtor*^+/+ or +/fl^ and *Foxp3*^Cre^*Mtor*^fl/fl^ mice. Error bars show mean ± s.e.m. *P < 0.05; **P < 0.01; ***P < 0.001; ns, not significant; unpaired, two-tailed Student’s *t*-test. Data are representative of at least twelve (**a**, **b**, **e**) or three (**c**) biological replicates per group. Data are quantified from the numbers of mice as indicated in the legend key (**b**, **d**) or from ten or eleven (**f**, IFN-γ^+^ and IL-17A^+^ cells from *Foxp3*^Cre^*Mtor*^+/+ or +/fl^ or *Foxp3*^Cre^*Mtor*^fl/fl^ mice, respectively), nine or ten (**f**, IL-4^+^ cells from *Foxp3*^Cre^*Mtor*^+/+ or +/fl^ or *Foxp3*^Cre^*Mtor*^fl/fl^ mice, respectively), eight (**g**), or nine (**h**) biological replicates per group, compiled from more than eight independent experiments (**f**−**h**). Numbers indicate percentage of cells in gates

To determine the effects of long-term deletion of *Mtor* on T_reg_-cell suppressive function in vivo, we next generated mice bearing a conditional deletion of *Mtor* within all committed Foxp3^+^ T_reg_ cells (denoted as *Foxp3*^Cre^*Mtor*^fl/fl^ mice). As anticipated, *Mtor* was efficiently deleted within Foxp3-YFP^+^ T_reg_ cells from *Foxp3*^Cre^*Mtor*^fl/fl^ mice (Supplementary Fig. 1a). In contrast to their littermate controls that remained healthy, *Foxp3*^Cre^*Mtor*^fl/fl^ mice developed an early-onset lymphoproliferative and autoimmune disease, indicated by reduced body size and hunched posture, enlargement of peripheral lymphoid organs, and extensive lymphocyte and/or myeloid cell infiltration in multiple organs, such as the skin and lung (Fig. 2a–c). This disease ultimately led to the early death of *Foxp3*^Cre^*Mtor*^fl/fl^ mice (Fig. 2d). These mice had reduced frequencies of CD44^lo^CD62L^hi^ naive CD4^+^ and CD8^+^ T cells and increased frequencies of CD44^hi^CD62L^lo^ effector/memory phenotype CD4^+^ and CD8^+^ T cells (Fig. 2e). There were also significant increases in IFN-γ-, IL-4-, IL-10-, IL-13-, and IL-17A-producing CD4^+^ T cells and IFN-γ-producing CD8^+^ T cells in mice with mTOR-deficient T_reg_ cells (Fig. 2f and Supplementary Fig. 1b). *Foxp3*^Cre^*Mtor*^fl/fl^ mice had 5–10-fold and 10–15-fold increases in the frequencies of cells producing T_H_2- or T_H_17-associated cytokines, respectively, while IFN-γ-producing cells were increased by ~5-fold (Supplementary Fig. 1c). Within T_reg_ cells, the frequency of IFN-γ-producing cells was also increased in *Foxp3*^Cre^*Mtor*^fl/fl^ mice (Supplementary Fig. 1d). We also found that the frequencies and total numbers of PD-1^+^CXCR5^+^ T_FH_ cells (Fig. 2g) and CD95^+^GL7^+^ GC B cells (Fig. 2h) were increased in *Foxp3*^Cre^*Mtor*^fl/fl^ mice. We, therefore, performed immunohistochemistry analysis of GCs, B cells, and T cells in whole tissue sections. This analysis revealed that PNA^+^ cells were diffusely distributed in extrafollicular regions, while T and B cells were markedly increased, in mesenteric lymph nodes (Supplementary Fig. 1e). *Foxp3*^Cre^*Rptor*^fl/fl^ mice, which have impaired mTORC1 signaling^30^, also had elevated T_FH_ and GC B-cell responses (Supplementary Fig. 1f), indicating that mTORC1 is essential for the T_reg_-cell-mediated suppression of spontaneous GC reactions. To determine if T_FH_ cells produce elevated levels of IL-4 and/or IL-21 to promote GC reactions^35,36^, we isolated CD4^+^Foxp3-YFP^−^CD44^hi^CXCR5^−^PD-1^−^ non-T_FH_ cells and CD4^+^Foxp3-YFP^−^CD44^hi^CXCR5^+^PD-1^+^ T_FH_ cells from *Foxp3*^Cre^*Mtor*^fl/fl^ mice and their littermate controls, and measured the expression of *Il4* and *Il21*. T_FH_ cells from *Foxp3*^Cre^*Mtor*^fl/fl^ mice had increased expression of *Il4*, but not *Il21*, while non-T_FH_ cells had increased expression of both *Il4* and *Il21* (Supplementary Fig. 1g, h). Thus, constitutive depletion of mTOR revealed its essential role for T_reg_ cell-mediated suppression of conventional T-cell responses in vivo.

### mTOR supports T_reg_-cell suppression of mucosal T_H_2 responses

**Fig. 3:**
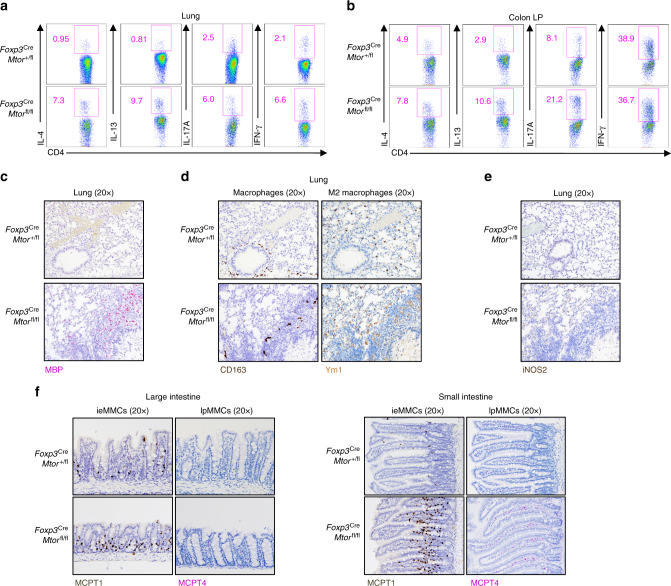
T_reg_ cells require mTOR for the suppression of mucosal T_H_2 responses. **a**, **b** Flow cytometry analysis of cytokine-producing CD4^+^ T cells isolated from the lung (**a**) or colon lamina propria (LP) (**b**) of *Foxp3*^Cre^*Mto*^+/fl^ or *Foxp3*^Cre^*Mtor*^fl/fl^ mice. **c** Representative images of major basic protein (MBP) staining for eosinophils in the lung of *Foxp3*^Cre^*Mtor*^+/fl^ and *Foxp3*^Cre^*Mtor*^fl/fl^ mice. **d** Representative images for M2 macrophages in the lung of *Foxp3*^Cre^*Mtor*^+/fl^ or *Foxp3*^Cre^*Mtor*^fl/fl^ mice by CD163 and Ym1 staining. **e** Representative images for neutrophils, as indicated by inducible nitric oxide synthease 2 (iNOS2) staining, in the lung of *Foxp3*^Cre^*Mtor*^+/fl^ and *Foxp3*^Cre^*Mtor*^fl/fl^ mice. **f** Representative immunohistochemistry of ieMMCs and lpMMCs in the large intestines (left) and small intestines (right) of *Foxp3*^Cre^*Mtor*^fl/+^ or *Foxp3*^Cre^*Mtor*^fl/fl^ mice. Data are representative of four independent experiments (**a**, **b**) or three biological replicates per group (**c**−**f**). Numbers indicate percentage of cells in gates

T_reg_ cells regulate T-cell responses important for tissue homeostasis, especially at barrier surfaces like the lung, intestines, and skin^1,2^. We found that in the lung of *Foxp3*^Cre^*Mtor*^fl/fl^ mice, there were respective 5–10-fold and 10–15-fold increases of IL-4- and IL-13-producing CD4^+^ T cells, while the increases in T_H_1 and T_H_17 responses were less pronounced (2–3-fold increased) (Fig. 3a and Supplementary Fig. 2a). T_H_2 and T_H_17 responses were also more elevated than T_H_1 responses in the colon lamina propria (Fig. 3b). Because we found a consistent increase of T_H_2 cytokines in both the lung and colon lamina propria and acute deletion of mTOR led to a profound increase of T_H_2 responses (Fig. 1h), we next performed comprehensive immunohistochemistry analyses of multiple organs in *Foxp3*^Cre^*Mtor*^fl/fl^ mice. Elevated T_H_2 responses are associated with an accumulation of eosinophils, alternatively activated M2 macrophages, and neutrophils in target tissues^16^. Indeed, MBP^+^ eosinophils were increased in the lung (Fig. 3c), as well as the dermis of the skin (Supplementary Fig. 2b) of mice-bearing mTOR-deficient T_reg_ cells. Additionally, CD163^+^ macrophages were expanded, including Ym1^+^ M2 macrophages present in the alveolar space and interstitium of the lung (Fig. 3d). Increased M2 macrophage activation was also evident in the skin (Supplementary Fig. 2c). We also observed an increase of cells positive for iNOS2, which primarily stains for neutrophils, in the lung (Fig. 3e) and skin (Supplementary Fig. 2d). T_H_2 inflammation is also associated with the accumulation of mucosal mast cells (MMCs) in the intestines^37^. *Foxp3*^Cre^*Mtor*^fl/fl^ mice had an increase of MCPT1^+^ interepithelial MMCs and MCPT4^+^ lamina propria MMCs in the large and small intestines (Fig. 3f). Altogether, these results underscore an important role for mTOR in mediating T_reg_-cell-dependent suppression of effector T-cell responses, especially T_H_2-associated events, within mucosal tissues.

### mTOR enforces mucosal tT_reg_- and pT_reg_-cell homeostasis

**Fig. 4:**
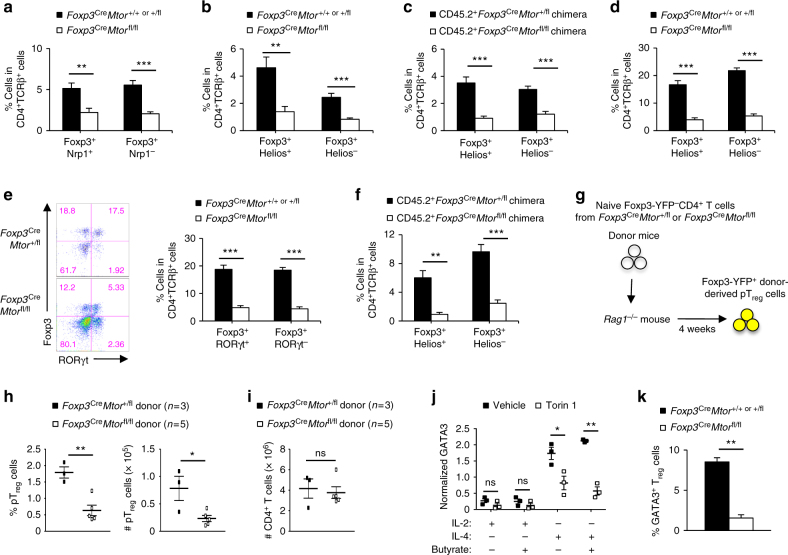
Mucosal tT_reg_- and pT_reg_-cell homeostasis is altered in the absence of mTOR. **a**, **b** Quantification of the frequencies of Nrp1^+^ and Nrp1^–^ (**a**) or Helios^+^ and Helios^–^ T_reg_ cells (**b**) in the lung of *Foxp3*^Cre^*Mtor*^+/+ or +/fl^ and *Foxp3*^Cre^*Mtor*^fl/fl^ mice, respectively. **c** Quantification of the frequencies of Helios^+^ and Helios^–^ T_reg_ cells among the CD45.2^+^CD4^+^TCRβ^+^ T cells in the lung of mixed bone marrow chimeras. **d** Quantification of the frequencies of Helios^+^ and Helios^–^ T_reg_ cells in the colon lamina propria of *Foxp3*^Cre^*Mtor*^+/+ or +/fl^ and *Foxp3*^Cre^*Mtor*^fl/fl^ mice. **e** Flow cytometry analysis of Foxp3 vs. RORγt expression (left) and quantification of the frequencies of RORγt^+^ and RORγt^–^ T_reg_ cells (right) in the colon lamina propria of *Foxp3*^Cre^*Mtor*^+/+ or +/fl^ and *Foxp3*^Cre^*Mtor*^fl/fl^ mice. **f** Quantification of the frequencies of Helios^+^ and Helios^–^ T_reg_ cells among the CD45.2^+^CD4^+^TCRβ^+^ T cells in the colon lamina propria of mixed bone marrow chimeras. **g** Experimental schematic for in vivo pT_reg_ maintenance assay. **h**, **i** Quantification of frequency and/or number of donor-derived Foxp3-YFP^+^ pT_reg_ cells (**h**) or total CD4^+^ T cells (**i**) in *Rag1*^–/–^ mice 4 weeks after adoptive transfer of naive T cells isolated from *Foxp3*^Cre^*Mtor*^+/fl^ and *Foxp3*^Cre^*Mtor*^fl/fl^ mice. **j** Quantification of GATA3 expression in T_reg_ cells stimulated under various conditions (with TGF-β and IL-6 included in all the conditions) for 3 days in the presence or absence of Torin 1. **k** Quantification of GATA3^+^ T_reg_ cells (Foxp3^+^GATA3^+^ in CD4^+^TCRβ^+^) from the colon lamina propria of *Foxp3*^Cre^*Mtor*^+/+ or +/fl^ and *Foxp3*^Cre^*Mtor*^fl/fl^ mice. Error bars show mean ± s.e.m. *P < 0.05; **P < 0.01; ***P < 0.001; ns, not significant; unpaired, two-tailed Student’s *t*-test. Data are quantified from five (**a**, **b**), eight (**c**), ten (**d**), eleven (**e**), seven (**f**), three or five (**h**, **i**; as indicated), three (**j**), or six (**k**) biological replicates, compiled from five (**a**, **b**), four (**c**, **f**), eight (**d**), nine (**e**), two (**h**, **i**), three (**j**), or six (**k**) independent experiments. Numbers indicate percentage of cells in quadrants

Recent work shows that tT_reg_ cells present in tissues exhibit T_H_2-biased gene signatures and express transcription factors essential for the suppression of T_H_2 responses^12,38,39^. Additionally, the absence of pT_reg_ cells drives elevated T_H_2 responses in mucosal tissues^16–18^. Therefore, we hypothesized that reduced abundance of tT_reg_ and/or pT_reg_ cells might account for increased T_H_2 responses in the lung and colon of *Foxp3*^Cre^*Mtor*^fl/fl^ mice. Neuropilin-1 (Nrp1) and Helios are expressed at higher levels in tT_reg_ than pT_reg_ cells^40,41^. We found that there was a significant decrease in the frequency of Nrp1^+^ tT_reg_ and Nrp1^−^ pT_reg_ cells in the lung of *Foxp3*^Cre^*Mtor*^fl/fl^ mice (Fig. 4a). Helios staining revealed a similar reduction in tT_reg_ and pT_reg_ cells in the lung of *Foxp3*^Cre^*Mtor*^fl/fl^ mice (Fig. 4b). We tested if these effects were cell-intrinsic by adoptively transferring an equal ratio of CD45.1^+^ wild-type bone marrow cells and CD45.2^+^ *Foxp3*^Cre^*Mtor*^+/fl^ or *Foxp3*^Cre^*Mtor*^fl/fl^ bone marrow cells into irradiated *Rag1*^–/–^-recipient mice. This inflammation-free system confirmed that the reduction of these lung T_reg_-cell populations was cell-intrinsic (Fig. 4c). We next examined pT_reg_ and tT_reg_ cell populations in the colon lamina propria of *Foxp3*^Cre^*Mtor*^fl/fl^ mice by staining for either Helios or RORγt, a transcription factor selectively enriched in pT_reg_ cells isolated from the intestines^17,19^. Similar to our observations in the lung, pT_reg_ cells, as well as Helios^+^ or RORγt^–^ tT_reg_ cells, were reduced in the colon lamina propria of *Foxp3*^Cre^*Mtor*^fl/fl^ mice (Fig. 4d, e). The reduction of RORγt^+^ pT_reg_ cells may also contribute to the increased T_H_17 cell activation in the colon lamina propria of *Foxp3*^Cre^*Mtor*^fl/fl^ mice (Fig. 3b)^19^. Analysis of Helios^+^ and Helios^−^ T_reg_-cell populations in the colon lamina propria from mixed bone marrow chimeras verified cell-intrinsic effects (Fig. 4f). Altogether, these results indicate that the accumulation of mucosal tissue tT_reg_ and pT_reg_ cells is disrupted in the absence of mTOR.

To determine the role for mTOR in pT_reg_-cell maintenance in vivo, we purified naive Foxp3-YFP^−^CD4^+^ T cells from either *Foxp3*^Cre^*Mtor*^+/fl^ or *Foxp3*^Cre^*Mtor*^fl/fl^ mice and adoptively transferred these cells into *Rag1*^*–/–*^ mice (Fig. 4g). In this system, naive T cells can acquire Foxp3 expression^42^, and the concomitant expression of the Cre transgene induces *Mtor* deletion in pT_reg_ cells generated in vivo. The frequency and number of mTOR-deficient pT_reg_ cells were reduced in mesenteric lymph nodes (Fig. 4h), while the numbers of donor-derived total CD4^+^ T cells were comparable (Fig. 4i). These results indicate that mTOR promotes the maintenance of pT_reg_ cells in vivo.

Activated T_reg_ cells express GATA3, which is required to suppress T_H_2 responses^1,2,11,12,16,38^. Therefore, we next examined if mTOR regulates GATA3 expression in activated T_reg_ cells. We established an in vitro system where T_reg_ cells from wild-type mice were stimulated with anti-CD3 and anti-CD28 antibodies in the presence of TGF-β and IL-6 to mimic the environmental signals at mucosal sites^1,2,12^. As expected, compared with IL-2 stimulation, IL-4 strongly upregulated GATA3 expression under these conditions^12^. However, IL-4-induced GATA3 upregulation was diminished upon inhibition of mTOR activity (Fig. 4j). The frequency of GATA3^+^ T_reg_ cells was also significantly reduced in the colon lamina propria (Fig. 4k), a site where these cells are enriched under steady state^11,12^. These in vitro and in vivo results highlight the requirement of mTOR signaling for GATA3 expression in T_reg_ cells.

### mTOR promotes eT_reg_-cell generation

**Fig. 5:**
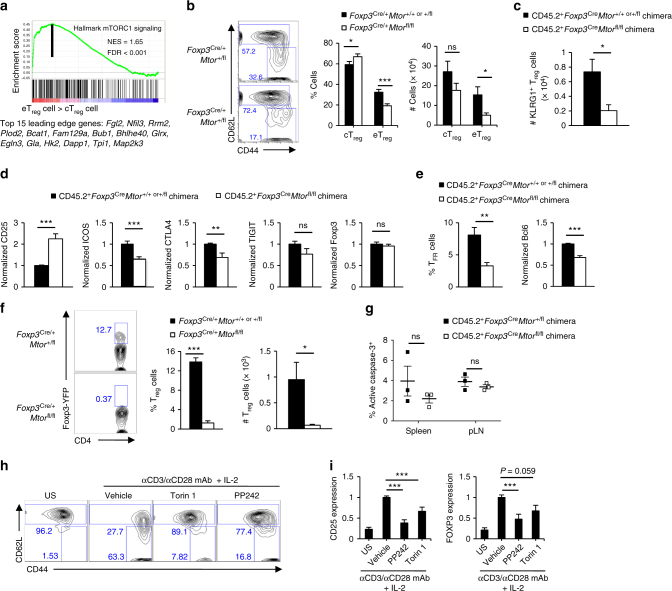
mTOR is essential for eT_reg_ cell differentiation. **a** Enrichment plot of the Hallmark mTORC1 pathway activated in eT_reg_ cells compared to cT_reg_ cells, identified by gene set enrichment analysis (GSEA). The top 15 enriched genes (position indicated by the vertical black line) are listed below the plot. **b** Flow cytometry analysis (left) and quantification of frequencies and cell numbers (right) of CD4^+^Foxp3-YFP^+^CD44^lo^CD62L^hi^ cT_reg_ cells and CD4^+^Foxp3-YFP^+^CD44^hi^CD62L^lo^ eT_reg_ cells in *Foxp3*^Cre/+^*Mtor*^*+/+* or +/fl^ and *Foxp3*^Cre/+^*Mtor*^fl/fl^ mosaic mice. **c** Quantification of the number of KLRG1^+^ T_reg_ cells in the spleen of mixed bone marrow chimeras. **d** Quantification of CD25, ICOS, CTLA4, TIGIT, and Foxp3 expression in T_reg_ cells from mixed bone marrow chimeras. **e** Quantification of the frequency of T_FR_ cells (CD4^+^Foxp3-YFP^+^CXCR5^+^PD-1^+^ T_reg_ cells, left) and Bcl6 expression in total Foxp3^+^ T_reg_ cells (right) in mixed bone marrow chimeras. **f** Flow cytometry analysis (left) and quantification of the frequency and number (right) of Foxp3-YFP^+^ T_reg_ cells in the colon lamina propria of *Foxp3*^Cre/+^*Mtor*^*+/+* or +/fl^ and *Foxp3*^Cre/+^*Mtor*^fl/fl^ mosaic mice. **g** Quantification of active caspase-3 in CD45.2^+^CD4^+^Foxp3-YFP^+^ T_reg_ cells from mixed bone marrow chimeras. **h** Flow cytometry analysis of CD44 vs. CD62L expression on cT_reg_ cells activated under the indicated conditions for 3 days. US: unstimulated. **i** Quantification of CD25 and FOXP3 expression in human CD4^+^CD25^+^CD45RA^+^CD45RO^–^ naive T_reg_ cells activated for 3 days in the presence or absence of Torin 1 or PP242. Error bars show mean ± s.e.m. *P < 0.05; **P < 0.01; ***P < 0.001; ns, not significant; unpaired, two-tailed Student’s *t*-test. Data are representative of at least six (**b**), six (**f**), or three (**h**) biological replicates per group or are quantified from eleven (**b**), fifteen (**c**), seven or eight (**d**; CD45.2^+^*Foxp3*^Cre^*Mtor*^+/+ or +/+^ chimera or CD45.2^+^*Foxp3*^Cre^*Mtor*^+/+ or +/fl^ mice, respectively; CD25 and Foxp3), ten (**d**; ICOS, CTLA4, and TIGIT; **e**), six (**f**), three (**g**), or five (**i**) biological replicates, compiled from seven (**b**), eight (**c**), four (**d**, TIGIT; **f**), five (**d**; CD25 and Foxp3), six (**d**; ICOS and CTLA4; **e**), or two (**g**, **i**) independent experiments. Numbers indicate percentage of cells in gates

After thymic development, peripheral cT_reg_ cells undergo antigen and inflammation-driven activation and differentiate into eT_reg_ cells that are enriched in tissues, including the lung and colon lamina propria^1,2,7,8,21,22^. Although eT_reg_ cells are crucial for immune homeostasis, the molecular requirements driving their activation and function are still poorly defined. Our above data indicated that mTOR-deficient tT_reg_ cells were reduced in the lung and colon. Moreover, unbiased GSEA showed that mTORC1 signaling was enriched in CD44^hi^CD62L^lo^ eT_reg_ cells compared to CD44^lo^CD62L^hi^ cT_reg_ cells (Fig. 5a). Given these results, we next tested whether mTOR regulates eT_reg_-cell generation. Because T_reg_ cells isolated from inflammatory environments could undergo secondary phenotypic changes, we analyzed cell-intrinsic effects of mTOR deficiency in cT_reg_ and eT_reg_ cells isolated from healthy, female mosaic mice (designated as *Foxp3*^Cre/+^). There was an increase in the frequency but not number of Foxp3-YFP^+^CD44^lo^CD62L^hi^ cT_reg_ cells and a reduction in the frequency and number of Foxp3-YFP^+^CD44^hi^CD62L^lo^ eT_reg_ cells in the spleen of *Foxp3*^Cre/+^*Mtor*^fl/fl^ mosaic mice (Fig. 5b). We also confirmed the reduction of eT_reg_ cells in the spleen of mixed bone marrow chimeras (Supplementary Fig. 3a). Consistent with elevated mTORC1 signaling in eT_reg_ cells (Fig. 5a), the frequency and number of eT_reg_ cells were reduced in the absence of *Rptor* (Supplementary Fig. 3b). The number of KLRG1^+^ T_reg_ cells was also reduced in absence of mTOR, consistent with a reduction of eT_reg_ cells (Fig. 5c and Supplementary Fig. 3c)^9^. The expression of CD25, a marker expressed at higher levels on cT_reg_ cells than eT_reg_ cells^9^, was increased on mTOR-deficient T_reg_ cells (Fig. 5d and Supplementary Fig. 3d). Moreover, eT_reg_-cell-associated molecules like ICOS and CTLA4 were expressed at lower levels in the absence of mTOR, while the expression of TIGIT or Foxp3 was equivalent between the control and mTOR-deficient T_reg_ cells (Fig. 5d and Supplementary Fig. 3d)^1,2,9^. Activated T_reg_ cells also differentiate into specialized or tissue-resident T_reg_-cell populations, including CXCR5^+^PD1^+^Foxp3^+^ T_FR_ cells that express Bcl6^5,10,13^. Both T_FR_ cells and Bcl6 expression were reduced in the absence of mTOR (Fig. 5e and Supplementary Fig. 3e). Consistent with our earlier analysis of mixed bone marrow chimeras, there was nearly a complete loss of colon T_reg_ cells in *Foxp3*^Cre^*Mtor*^fl/fl^ mosaic mice (Fig. 5f). Thus, mTOR is essential for maintaining eT_reg_ cells in vivo.

Mechanistically, the loss of eT_reg_ cells in the absence of mTOR could be due to defective survival or reduced activation-induced differentiation. To test the former, we analyzed the expression of active caspase-3 in control and mTOR-deficient T_reg_ cells isolated from mixed bone marrow chimeras and found normal survival of mTOR-deficient T_reg_ cells (Fig. 5g). Also, the frequency of 7AAD^+^ cells was similar or reduced in purified CD44^lo^CD62L^hi^ cT_reg_ cells activated in the presence of the mTOR inhibitors (Supplementary Fig. 3f), further indicating that mTOR is not essential for cell survival.

To test the role of mTOR in activation-induced differentiation, we purified CD44^lo^CD62L^hi^ cT_reg_ cells from wild-type mice and activated them for 3 days in the presence or absence of the mTOR inhibitors, Torin 1 and PP242^43^. We found that cT_reg_ cells differentiation into CD44^hi^CD62L^lo^ eT_reg_-like cells was impaired by mTOR inhibition (Fig. 5h). Similarly, the frequency of mTOR-deficient T_reg_ cells in the spleen and peripheral lymph nodes of DT-treated *Foxp3*^Cre/DTR^*Mtor*^fl/fl^ mice was reduced relative to the controls (Supplementary Fig. 3g, left panel), but total numbers of T_reg_ cells were not significantly different (Supplementary Fig. 3g, right panel), likely due to the increased organ size (Fig. 1e). Thus, mTOR-deficient T_reg_ cells also fail to appropriately respond to activation-induced signals in vivo. We also investigated if these regulatory pathways applied to human cells. In the presence of the mTOR inhibitors, activated human CD45RA^hi^CD45RO^lo^ naive T_reg_ cells had impaired upregulation of CD25 and FOXP3 (Fig. 5i), which are expressed more abundantly in human CD45RA^lo^CD45RO^hi^ activated T_reg_ cells than naive T_reg_ cells^44^. Thus, mTOR activity represents an evolutionarily conserved pathway for driving eT_reg_-cell generation.

### mTOR links activation signals to IRF4 upregulation

**Fig. 6:**
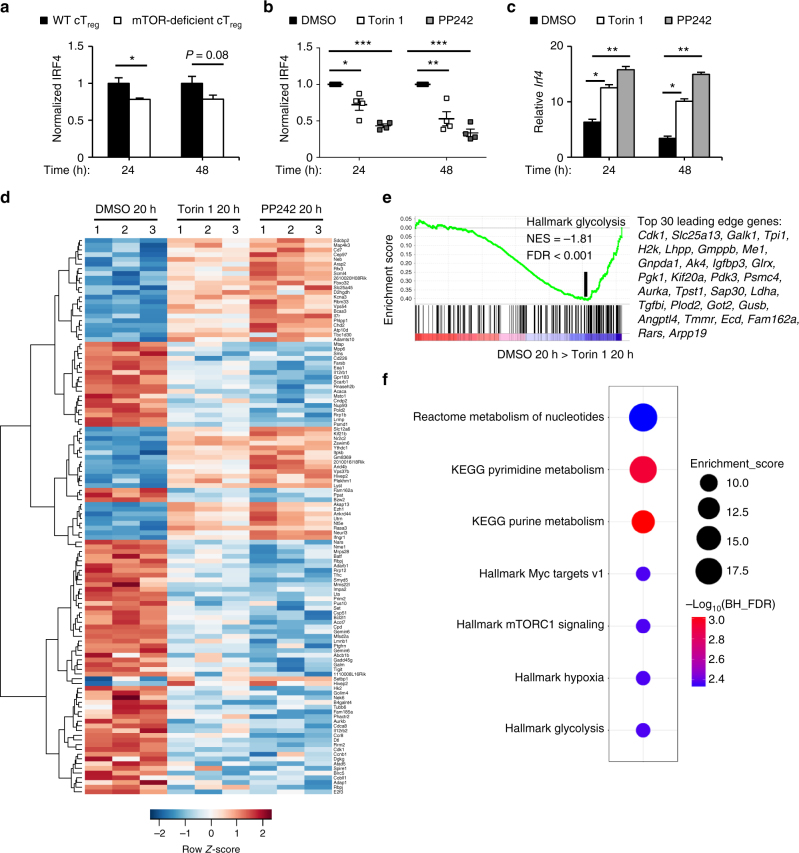
mTOR links activation signals to upregulation of IRF4 expression and downstream targets. **a** Wild-type (WT) and mTOR-deficient CD4^+^Foxp3-YFP^+^CD44^lo^CD62L^hi^ cT_reg_ cells were purified from mixed bone marrow chimeras and activated under the indicated conditions for 24 and 48 h. IRF4 expression was assessed by flow cytometry. **b** CD4^+^Foxp3-YFP^+^CD44^lo^CD62L^hi^ cT_reg_ cells were purified from *Foxp3*^Cre^ mice and activated as in **a** in the presence or absence of Torin 1 and PP242. IRF4 expression was assessed by flow cytometry. **c** cT_reg_ cells were purified and activated as in **b** and *Irf4* mRNA expression was analyzed by qPCR. **d** Heat map of IRF4 target genes differentially expressed in resting cT_reg_ cells or cT_reg_ cells activated in the presence of DMSO, Torin 1, or PP242 for 20 h. **e** Enrichment plot of the Hallmark glycolysis pathway in cT_reg_ cells activated for 20 h in the presence of either Torin 1 or DMSO control, identified by gene set enrichment analysis (GSEA). The top 30 enriched genes (position indicated by the vertical black line) are listed. **f** Functional enrichment of the IRF4 target genes shown in **d**. Error bars show mean ± s.e.m. *P < 0.05; **P < 0.01; ***P < 0.001; ns, not significant; unpaired, two-tailed Student’s *t*-test. Data are representative of three independent experiments (**b**) or are quantified from five (**a**) and four (**b**, **c**) biological replicates, compiled from two independent experiments (**a**, **c**)

IRF4 is induced by TCR signals to promote eT_reg_-cell differentiation and regulates T_reg_-cell-mediated suppression of T_H_2 responses in vivo^7,8,15,21,22^. To determine if mTOR induces IRF4 expression upon activation, we purified control and mTOR-deficient cT_reg_ cells and activated them for 24 and 48 h before analyzing IRF4 expression by flow cytometry. IRF4 expression was reduced at 24 and 48 h after activation (Fig. 6a). Acute mTOR inhibition with Torin 1 or PP242 also significantly reduced activation-induced upregulation of IRF4 in cT_reg_ cells (Fig. 6b). Mechanistically, mTOR controls IRF4 expression at the post-transcriptional level, because cT_reg_ cells activated in the presence of the mTOR inhibitors for 24 or 48 h had increased *Irf4* expression compared to the internal controls (Fig. 6c). To show that the ~20–30% reduction of IRF4 expression was biologically important, we analyzed gene expression profiles in cT_reg_ cells activated in the presence or absence of Torin 1 or PP242. Among the genes that were consistently altered by both inhibitors were 124 IRF4 target genes, including *Ccr8* and *Eea1*^8,15,21,22^ (Fig. 6d). Also, the loss of IRF4 expression likely accounted for the impairment of mTOR-deficient T_reg_ cells to express ICOS (Fig. 5d and Supplementary Fig. 3d), which is induced by IRF4-dependent mechanisms to drive eT_reg_-cell differentiation in vivo^9,15^. Altogether, these data indicate that mTOR promotes eT_reg_-cell differentiation, in part, by modulating IRF4 expression, which also helps explain how T_H_2 responses become elevated in *Foxp3*^Cre^*Mtor*^fl/fl^ mice.

### mTOR orchestrates mitochondrial metabolism in T_reg_ cells

Metabolism is a crucial determinant of T_reg_-cell biology^23^, but the mechanisms controlling metabolic rewiring required for the function of activated T_reg_ cells are not clear. GSEA showed that cT_reg_ cells upregulated mTORC1 signaling and several metabolic pathways, including glycolysis, upon activation (Supplementary Fig. 4a), while mTOR inhibitor-treated cT_reg_ cells had a significant downregulation of genes in the glycolytic pathway (Fig. 6e and Supplementary Table 1), including *Hk2* (Fig. 6d). Because IRF4 also promotes metabolic reprograming of conventional CD4^+^ and CD8^+^ T cells^45^, we determined if mTOR signals via IRF4 to regulate T_reg_-cell metabolism. We performed functional enrichment analysis of IRF4 targets that were differentially expressed in cT_reg_ cells activated in the presence of mTOR inhibitors (Fig. 6d). This analysis revealed enrichments for glycolytic and nucleotide metabolism and upstream regulators of these pathways, including Myc and mTORC1^30,46,47^ (Fig. 6f). Thus, the mTOR-IRF4 axis supports the upregulation of glycolytic and nucleotide metabolism during cT_reg_-cell activation.

**Fig. 7:**
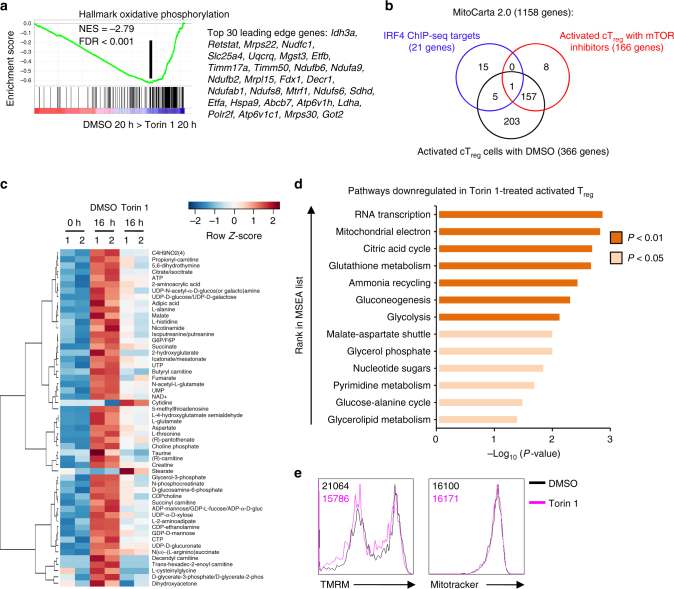
mTOR controls metabolic reprogramming upon T_reg_-cell activation. **a** Enrichment plot of the Hallmark oxidative phosphorylation pathway in cT_reg_ cells activated for 20 h in the presence of either Torin 1 or DMSO control, identified by gene set enrichment analysis (GSEA). The top 30 enriched genes (position indicated by the vertical black line) are listed. **b** Venn-diagram depicting mitochondria-related genes (defined in MitoCarta 2.0 database) that are IRF4 targets (blue circle), or differentially expressed in activated cT_reg_ cells (vs. unstimulated cells; black circle) or activated cT_reg_ cells treated with mTOR inhibitors (vs. those treated with DMSO; red circle). The numbers indicate the shared and independent genes in each category. **c** Heat map of differentially expressed intracellular metabolites in freshly isolated T_reg_ cells or T_reg_ cells activated for 16 h in the presence of DMSO or Torin 1. **d** Metabolite set enrichment analysis (MSEA) of KEGG metabolic pathways downregulated in Torin 1-treated activated T_reg_ cells compared to vehicle-treated activated T_reg_ cells. **e** Flow cytometry of TMRM and Mitotracker staining in T_reg_ cells activated for 20 h in the presence of Torin 1 or vehicle control. Data are representative of three (Mitotracker) or four (TMRM) biological replicates from two (Mitotracker) or three (TMRM) independent experiments (**e**)

Besides glycolysis, cT_reg_ cells upregulated genes in the oxidative phosphorylation pathway in an mTOR-dependent manner (Fig. 7a and Supplementary Table 1). Indeed, 366 mitochondrial genes (identified from the MitoCarta 2.0 database^48^) were differentially expressed in activated cT_reg_ cells (vs. unstimulated cells), and 158 of these genes were mTOR targets (Fig. 7b). Twenty-one mitochondrial genes were putative IRF4 gene targets (based on IRF4 ChIP-seq analysis^22^), but only six of these genes were induced upon cT_reg_-cell activation. Only one of these IRF4 targets (*Mrps28*) was upregulated in an mTOR-dependent manner during cT_reg_-cell activation (Fig. 7b). These data, combined with the functional enrichment analysis above, suggest that mTOR promotes mitochondrial gene expression in a largely IRF4-independent manner. To further test the effects of mTOR on the metabolic pathways, we performed metabolomics profiling using high-resolution mass spectrometry on resting and activated T_reg_ cells. We found that 54 metabolites were differentially expressed between T_reg_ cells activated in the presence of Torin 1 vs. DMSO (Fig. 7c). For instance, the expression of the TCA cycle intermediates isocitrate/citrate, malate, and succinate and the electron acceptor NAD^+^ were significantly decreased in T_reg_ cells activated in the presence of Torin 1 (Fig. 7c). Unbiased metabolite set enrichment analysis (MSEA) revealed that activated T_reg_ cells significantly upregulated metabolic pathways associated with mitochondria-dependent energy production and the biosynthesis of proteins and nucleotides, such as the citric acid cycle, the mitochondrial electron transport chain, and pyrimidine biosynthesis^49^ (Supplementary Fig. 4b). The upregulation of these mitochondria-related metabolic pathways was impaired when T_reg_ cells were activated in the presence of Torin 1 (Fig. 7d). Consistent with this observation, T_reg_ cells activated in the presence of Torin 1 had lower mitochondrial membrane potential (TMRM), whereas mitochondria number as indicated by Mitotracker staining was comparable (Fig. 7e). Thus, mTOR links activation signals to IRF4-dependent and -independent transcriptional programs to induce metabolic reprogramming during T_reg_-cell activation.

### Tfam is essential for eT_reg_-cell homeostasis and function

**Fig. 8:**
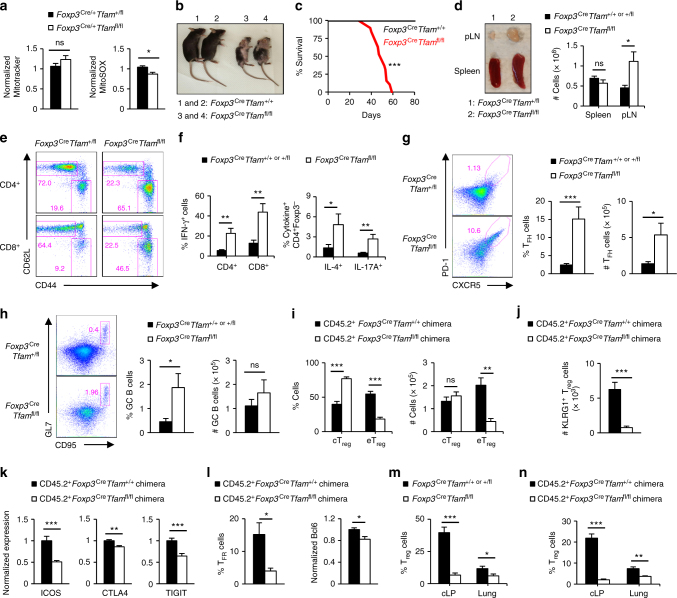
Mitochondrial metabolism is critical for T_reg_-cell function in vivo. **a** Quantification of Mitotracker (left) and MitoSOX (right) in T_reg_ cells from *Foxp3*^Cre/+^*Tfam*^+/fl^ and *Foxp3*^Cre/+^*Tfam*^fl/fl^ mosaic mice. **b** Image of 8-week-old mice. **c** Survival curve of *Foxp3*^Cre^*Tfam*^+/+^ (*n* = 14) and *Foxp3*^Cre^*Tfam*^fl/fl^ mice (*n* = 19). **d** Representative image of lymphadenopathy (left) and cell numbers in the spleen and peripheral lymph nodes (pLN) of *Foxp3*^Cre^*Tfam*^+/fl^ and *Foxp3*^Cre^*Tfam*^fl/fl^ mice. **e** Flow cytometry analysis of naive and effector/memory CD4^+^Foxp3-YFP^–^ (depicted as CD4^+^) or CD8^+^ T cells in *Foxp3*^Cre^*Tfam*^+/fl^ and *Foxp3*^Cre^*Tfam*^fl/fl^ mice. **f** Quantification of cytokine production by CD4^+^Foxp3^–^ and CD8^+^ T cells. **g** Flow cytometry analysis (left) and frequency and number (right) of T_FH_ cells. **h** Flow cytometry analysis (left) and frequency and number (right) of GC B cells. **i**, **j** Quantification of frequencies and numbers of cT_reg_ cells and eT_reg_ cells (**i**) or number of KLRG1^+^ T_reg_ cells (**j**) in the spleen of mixed bone marrow chimeras. **k** Quantification of ICOS, CTLA4, and TIGIT expression in T_reg_ cells from mixed bone marrow chimeras. **l** Quantification of the frequency of T_FR_ cells (left) and Bcl6 expression in total Foxp3^+^ T_reg_ cells (right) in mixed bone marrow chimeras. **m**, **n** Quantification of frequency of T_reg_ cells in the colon lamina propria and lung of *Foxp3*^Cre^*Tfam*^+/+ or +/fl^ or *Foxp3*^Cre^*Tfam*^fl/fl^ mice (**m**) or mixed bone marrow chimeras (**n**). Error bars show mean ± s.e.m. *P < 0.05; **P < 0.01; ***P < 0.001; ns, not significant; unpaired, two-tailed Student’s *t*-test. Data are representative of nine (**b**, **d**, **e**), twelve (**g**), or ten (**h**) biological replicates per group, or are quantified from five (**a**), eight or nine (**d**, **f**; *Foxp3*^Cre^*Tfam*^fl/fl^ or *Foxp3*^Cre^*Tfam*^+/+ or +/fl^ mice, respectively), twelve (**g**), ten (**h**), nine or ten (**i**−**l**, **n**; CD45.2^+^*Foxp3*^Cre^*Tfam*^+/+^ chimeras and CD45.2^+^*Foxp3*^Cre^*Tfam*^+/fl^ chimeras, respectively), five (**m**, colon), or eight (**m**, lung) biological replicates per group, compiled from four (**a**), six (**d**, **f**), eight (**g**), seven (**h**), four (**i**−**l**, **n**), three (**m**; colon), or five (**m**; lung) independent experiments. Numbers indicate percentage of cells in gates

We next genetically defined the importance of mitochondrial metabolism in T_reg_-cell function in vivo. We conditionally deleted mitochondrial transcription factor A (Tfam), a nuclear-encoded transcription factor essential for efficient electron transport chain activity^50,51^, in T_reg_ cells by breeding *Foxp3*^Cre^ transgenic mice with mice-bearing floxed alleles for *Tfam*^51^. Tfam-deficient T_reg_ cells isolated from *Foxp3*^Cre/+^*Tfam*^fl/fl^ mosaic mice had comparable mitochondrial content but less mitochondria-derived reactive oxygen species (ROS) (Fig. 8a), consistent with reduced mitochondrial respiratory chain function. We found that Tfam is critical for T_reg_-cell function, as *Foxp3*^Cre^*Tfam*^fl/fl^ mice developed a severe inflammatory disease associated with smaller body size, skin inflammation, alopecia (Fig. 8b), early lethality (Fig. 8c), and enlargement of the peripheral lymph nodes (Fig. 8d). Further, there were increased effector/memory T cells (Fig. 8e) and significantly enhanced IFN-γ-producing CD8^+^ T cell and T_H_1, T_H_2, and T_H_17-cell activation (Fig. 8f) in diseased mice-bearing Tfam-deficient T_reg_ cells. Tfam-deficient T_reg_ cells also had a propensity for increased IFN-γ production (Supplementary Fig. 5a). Moreover, the frequency and number of T_FH_ cells were increased (Fig. 8g). Only the frequency, not the number, of GC B cells was increased, likely due to a reduction of total B220^+^ B cells in *Foxp3*^Cre^*Tfam*^fl/fl^ mice (Fig. 8h and Supplementary Fig. 5b). Thus, Tfam deficiency in T_reg_ cells leads to altered immune homeostasis and development of autoimmunity.

We next analyzed T_reg_-cell populations in mixed bone marrow chimeras to determine the cell-intrinsic role of Tfam-dependent mitochondrial metabolism in eT_reg_-cell accumulation and homeostasis. There was a reduction in the frequency and number of CD44^hi^CD62L^lo^ eT_reg_ cells (Fig. 8i) and KLRG1^+^ T_reg_ cells (Fig. 8j) in the absence of Tfam. Further, Tfam-deficient T_reg_ cells had reduced expression of ICOS and CTLA4 (Fig. 8k), but not Foxp3 (Supplementary Fig. 5c). However, unlike mTOR-deficient T_reg_ cells, CD25 expression was not increased on Tfam-deficient T_reg_ cells (Supplementary Fig. 5c), and TIGIT expression was reduced (Fig. 8k). Tfam deficiency also reduced T_FR_-cell generation and Bcl6 expression (Fig. 8l). Further, there was a cell-intrinsic reduction of Tfam-deficient T_reg_ cells within the colon lamina propria and the lung (Fig. 8m, n). Collectively, these results indicate that Tfam-dependent mitochondrial metabolism is critical for the function and homeostasis of activated T_reg_ cells in vivo.

## Discussion

Activated tT_reg_ and pT_reg_ cells are crucial for peripheral T-cell tolerance and tissue homeostasis. Here, we show that activated T_reg_-cell populations have increased mTOR signaling necessary for T_reg_-cell activation and tissue T_reg_-cell homeostasis. Mechanistically, mTOR tunes IRF4-dependent transcriptional programming and mitochondrial metabolism. In the absence of mTOR, activated tT_reg_ and pT_reg_ cells are severely decreased in mucosal tissues, associated with excessive T_H_2, and to a lesser extent T_H_1 and T_H_17 responses, and disrupted tissue homeostasis. Further, the homeostasis and suppressive activity of activated T_reg_ cells is impaired by the loss of mitochondrial metabolism and consequently leads to autoimmunity (Supplementary Fig. 5d). Thus, our data identify and establish a critical mTOR-dependent metabolic node that regulates the homeostasis and suppressive function of activated T_reg_ cells in vivo.

TCR-dependent signals coupled with co-stimulation and inflammatory cues drive T_reg_-cell activation and differentiation into specialized or tissue-resident T_reg_-cell subsets^1,2,7–9,21,22^. Several transcriptional programs are essential for the differentiation and function of activated T_reg_ cells^7,20–22,43,52,53^. However, the upstream signaling pathways driving eT_reg_-cell homeostasis are unknown. Here, we show that activation signals through mTOR are required for T_reg_-cell activation and function, thereby establishing the first kinase pathway, to our knowledge, that links TCR signals and transcriptional programs necessary for T_reg_-cell activation. Mechanistically, mTOR promotes the expression of IRF4 and GATA3, transcription factors that are essential for T_reg_-cell-dependent suppression of T_H_2 responses^7,11,12,15^. Moreover, IRF4 also enforces eT_reg_-cell differentiation and pT_reg_-cell homeostasis to limit mucosal T_H_2 responses^16,17,20–22^. Therefore, by promoting the expression of IRF4 and GATA3, mTOR maintains activated T_reg_-cell populations that facilitate tissue homeostasis. The mTOR-dependent induction of IRF4 expression in cT_reg_ cells was not transcriptionally regulated, and, in conventional CD4^+^ T cells^54^, occurs independently of the mTOR-4EBP1 translation axis. Therefore, mTOR likely regulates IRF4 expression at the post-translational level, such as via SUMOlyation^55^. A key question that remains is, why are mucosal tissues more sensitive to the upregulation of T_H_2 responses than secondary lymphoid organs in mice-bearing mTOR-deficient T_reg_ cells? One possibility is that these sites are enriched for activated tT_reg_- and pT_reg_-cell populations and hence their loss more readily increases T_H_2 responses at these sites than in the peripheral lymphoid organs^1,2,56^. Further, recent work shows that tissue T_reg_ cells express high levels of ST2 (IL-33 receptor)^38,39^, which induces GATA3 activation that biases T_reg_ cells toward the T_H_2 suppressive program^39^. Thus, mTOR deficiency and other conditions that decrease GATA3 expression will impair this feed forward loop and disrupt T_H_2-like T_reg_-cell suppressive responses.

Metabolic reprogramming contributes to cell fate decisions. However, the metabolic programs promoting the homeostasis and function of activated T_reg_ cells are not completely understood^23^. Despite the previous work suggesting an inhibitory role of mTOR for mitochondrial oxidative metabolism in induced T_reg_ cells in vitro^27^, we show here that mitochondrial metabolism is highly induced during T_reg_-cell activation in an mTOR-dependent manner, and is essential for activated T_reg_-cell function and tissue homeostasis in vivo. Indeed, T_reg_-specific deletion of *Tfam* impairs T_reg_-cell function, leading to the hyperactivation of conventional T cells and autoimmunity. Mechanistically, mitochondrial metabolism could affect eT_reg_-cell proliferation or survival within tissues as has been reported in vitro^27,28^. Of note, Raptor-deficient T_reg_ cells have reduced mitochondria-related gene expression^30^, and *Foxp3*^Cre^*Tfam*^fl/fl^ and *Foxp3*^Cre^*Rptor*^fl/fl^ mice have similar elevations in activated T-cell responses, disease pathologies, and survival kinetics^30^. Additionally, deficiency of Tfam and Raptor impairs T_FR_ cell accumulation^57^. Thus, our data suggest a key role for Raptor-mTORC1-induced mitochondrial metabolism in establishing the fate and function of activated T_reg_ cells in different microenvironments.

T_reg_ cells must adapt to different environmental cues to acquire unique suppressive functions, and the appropriate balance of mTOR and metabolic signaling appears to be linked to this process. Inactivation of mTORC1 alone or combined with mTORC2 disrupts T_reg_-cell suppressive activity^30^; however, whether mTORC1-independent functions of Raptor^58^ or Raptor/Rictor-independent mTOR complexes play roles in T_reg_-cell biology remained unclear. Our results here suggest that mTOR does act through Raptor and Rictor to promote T_reg_-cell function. Compared to Raptor deficiency alone, loss of mTOR or Raptor and Rictor in T_reg_ cells leads to similar extensions in lifespan^30^, which may be due to more limited tissue damage, such as in the intestines. The balance of mTORC1- and/or mTORC2-induced metabolic programs may also tune T_reg_-cell suppression of effector T-cell responses. For instance, Raptor-deficient T_reg_ cells have increased mTORC2-Akt activity^30^, which can upregulate glycolysis at the expense of mitochondrial metabolism and lead to inappropriate suppression of T_H_1 and T_FH_ responses^59–61^. Despite being dispensable for T_reg_-cell suppressive activity^30,61^, mTORC2 can promote T_reg_-cell trafficking to sites of inflammation and non-lymphoid tissues via upregulating glycolysis or suppressing Foxo1 activity^43,62^. Thus, gain of mTORC1 and concomitant loss of mTORC2 activity could reduce T_reg_-cell trafficking to T_H_1 and T_H_17 inflammatory sites^63–65^. mTOR may also promote trafficking to sites of T_H_2 inflammation by modulating *Ccr8* expression and/or CCL22/CCR4-induced chemotaxis^62,66^. We, therefore, propose that T_reg_-cell function is finely tuned by graded nature of mTOR signaling and metabolic programs, which are likely influenced by local environmental signals. This tunable nature of mTOR signaling in T_reg_ cells may offer a therapeutic strategy to modulate T_reg_-cell responses to selectively alter the conventional T-cell responses in autoimmunity, infectious diseases, and cancer.

## Methods

### Mice

C57BL/6, CD45.1^+^, *Cd4*^Cre^, *Foxp3*^DTR^*, Rag1*^*–/–*^, *Mtor*^fl^, and *Tfam*^fl^ mice were purchased from The Jackson Laboratory. *Foxp3*^Cre^ mice, from Dr. Alexander Rudensky, have been described previously^33^. All genetic models used in this study were on the C57BL/6 background, and both male and female mice were used for quantification and analysis, except for histological analysis where only male mice were used. Mice were generally 4–6-weeks-old unless otherwise indicated. The number of animals in each group are provided in the figures and/or figure legends. All mice were kept in specific pathogen-free conditions within the Animal Resource Center at St. Jude Children’s Research Hospital. The animal protocols were approved by the Institutional Animal Care and Use Committee of St. Jude Children’s Research Hospital. Mixed bone marrow chimeras were generated by adoptive transfer of CD45.1^+^ bone marrow cells mixed 1:1 with CD45.2^+^ bone marrow cells from *Foxp3*^Cre^*Mtor*^+/fl^ or *Foxp3*^Cre^*Mtor*^fl/fl^ mice into sub-lethally irradiated *Rag1*^*–/–*^ mice as described^30^. For the pT_reg_ cell in vivo maintenance model, CD4^+^Foxp3-YFP^−^CD44^lo^CD62L^hi^ naive T cells from the spleens and peripheral lymph nodes of *Foxp3*^Cre^*Mtor*^+/fl^ or *Foxp3*^Cre^*Mtor*^fl/fl^ mice were purified on a Synergy or Reflection fluorescence activated cell sorter (Sony Biotechnology). Then, 0.75 × 10^6^ cells were transferred via retroorbital injection into sex-matched *Rag1*^–/–^ mice. The presence of Foxp3-YFP^+^ T_reg_ cells was evaluated in the mesenteric lymph nodes 4 weeks later^42^. *Foxp3*^Cre/DTR^ mosaic mice were treated with DT (50 μg kg^−1^) i.p. three times per week, for a total of four injections. The mice were euthanized, and tissues were harvested for flow cytometry analysis 11 days following the first DT treatment. Sample sizes were chosen based upon previous data generated within the laboratory and were selected to maximize the chance of uncovering statistically significant differences of the mean. No animals were excluded from analysis.

### Flow cytometry

Lymphocytes were harvested form the peripheral lymphoid tissues by manual disruption or the colon lamina propria as previously described^30,67^. For surface marker analyses, cells were stained in PBS containing 2% (wt/vol) BSA and the appropriate antibodies. The following fluorescent-conjugate-labeled antibodies, purchased from various commercial sources (Biolegend, BD Biosciences, Thermo Fisher Scientific, and Sony Biotechnology), were used: anti-CD4 (clone RM4-5), anti-CD8 (clone 53-6.7), anti-B220 (clone RA3-6B2), anti-CD62L (clone MEL-14), anti-CD44 (clone IM7), anti-CD95 (clone Jo2), anti-GL7 (clone GL-7), anti-CD279 (PD-1) (Clone J43), and anti-TCRβ (clone H57-597) antibodies. Biotin-conjugated anti-CXCR5 antibody (clone 2G8) and PE-labeled streptavidin from BD Biosciences were used for T_FH_ cell staining. Intracellular staining was performed using the Foxp3/Transcription Factor Staining buffers (Cat #00-5523-00, Thermo Fisher Scientific) per the manufacturer’s instructions. The following antibodies were used: anti-CD152 (CTLA4) (clone UC10-4B9), anti-Foxp3 (clone NRRF-30), anti-RORγt (clone B2D), anti-GATA3 (clone TWAJ), anti-IRF4 (clone 3E4), anti-Helios (clone 22F6), anti-IL-4 (clone 11B11), anti-IL-10 (clone JES5-16E3), anti-IL-13 (clone eBio13A), anti-IFN-γ (clone XMG1.2), anti-IL-17A (clone TC11-18H10.1), anti-human CD25 (clone BC96), anti-human CD45RA (clone HI100), anti-human CD45RO (clone UCHL1), anti-human CD4 (clone A161A1), and anti-human FOXP3 (clone 236 A/E7). For intracellular cytokine staining, total splenocytes were stimulated for 4–5 h with phrobol 12-myristate 13-acetate (PMA) and ionomycin in the presence of monensin (BD Biosciences). Surface and intracellular staining was then performed as above. For active caspase-3 staining, surface molecules were stained before cells were fixed, permeabilized, and stained for intracellular active caspase-3 using the BD Biosciences active caspase-3 apoptosis kit per the manufacturer’s instructions (Cat # 550914). Staining for mitochondrial dyes (MitoSOX, TMRM, and Mitotracker Deep Red; Thermo Fisher Scientific) was performed as previously described^30^.

### Histology and immunohistochemistry

Tissues were fixed in 10% (vol/vol) neutral buffered formalin solution, embedded in paraffin, section, and stained with hematoxylin and eosin. Blinded samples were analyzed by an experienced pathologist (P.V.) for the presence of lesions indicative of autoimmune disease. For the identification of ieMMCs or lpMMCs, tissue sections from the small intestines and large intestines were respectively stained with primary rat anti-MCPT1 monoclonal antibody (Cat # 14-55303-82, Thermo Fisher Scientific) or goat anti-MCPT4 antibody (LS-B5958, LifeSpan Biosciences) as described previously^37^. GCs were identified by staining with anti-CD3 antibody and peanut agglutinin as described^61^.

### In vitro T_reg_-cell suppression assays

For analysis of T_reg_-cell suppression in vitro, CD4^+^CD25^hi^ T_reg_ cells or CD4^+^Foxp3-YFP^+^ T_reg_ cells, isolated from the lymphoid organs of the respective *Cd4*^Cre^- or *Foxp3*^Cre^-expressing mice, were co-cultured with naive CD4^+^ T cells and irradiated splenocytes as antigen presenting cells as previously described^30^. For suppression assays using in vitro activated T_reg_ cells, CD25^hi^ T_reg_ cells were sorted from the lymphoid organs of C57BL/6 mice, resuspended in complete Click’s medium containing IL-2 (200 U ml^−1^), and activated using anti-CD3 (10 μg ml^−1^) and anti-CD28 (10 μg ml^−1^) antibodies for 3 days in the presence of PP242 (500 nM, Tocris Bioscience) or vehicle control. The live cells were then isolated using Lymphocyte Separation medium and co-cultured with naive CD4^+^ T cells and irradiated splenocytes for 3 days, and the incorporation of [^3^H]-thymidine was assessed as described^30^.

### T_reg_-cell cultures

CD4^+^Foxp3-YFP^+^CD44^lo^CD62L^hi^ cT_reg_ cells were purified and activated with anti-CD3 and anti-CD28 antibodies in the presence of recombinant IL-2 for various times in the presence of vehicle, Torin 1 (50 nM) or PP242 (500 nM) before total RNA was harvested using the Qiagen RNeasy micro kit per the manufacturer’s instructions. Alternatively, cT_reg_ cells were stimulated as above for 1–3 days and analyzed by flow cytometry as previously described^43^. For analysis of GATA3 protein expression, CD4^+^CD25^+^ T_reg_ cells were isolated from the mesenteric lymph nodes using the CD25^+^ T_reg_-cell enrichment kit (Miltenyi). The cells were then activated with anti-CD3 (5 μg ml^−1^) and anti-CD28 (5 μg ml^−1^) antibodies for 3 days in the presence of various stimuli and/or Torin 1 (50 nM) as indicated in the figure: TGF-β (2 ng ml^−1^), IL-2 (200 U ml^−1^), IL-4 (20 ng ml^−1^), IL-6 (20 ng ml^−1^), and butyrate (125 μM). The expression of GATA3 in Foxp3^+^ T_reg_ cells was assessed by flow cytometry.

### Human T_reg_-cell cultures

All human studies were in compliance with the Declaration of Helsinki. Blood donors were recruited by the Blood Donor Center at St. Jude Children’s Research Hospital, where they provided written consent for their discarded blood products to be used for research. This consent form has been reviewed and approved by the Institutional Review Board at St. Jude Children’s Research Hospital. We were provided with apheresis rings containing peripheral blood mononuclear cells (PBMCs) isolated from de-identified donors. Human CD4^+^CD25^+^CD45RA^+^CD45RO^−^ naive T_reg_ cells were purified from these human PBMCs, and activated with anti-CD3 (clone OKT3, 5 μg ml^−1^) and anti-CD28 (clone CD28.2, 5 μg ml^−1^) for 3 days in the presence of IL-2 and mTOR inhibitors as above. The expression of human CD25 and human FOXP3 was then analyzed by flow cytometry.

### Metabolomics

CD4^+^CD25^hi^ T_reg_ cells, isolated from lymphoid organs of C57BL/6 mice, were purified and resuspended in complete Click’s medium. Then, 1.3 × 10^6^ T_reg_ cells were treated with medium alone or immobilized anti-CD3 antibody (10 μg ml^−1^) and anti-CD28 antibody (10 μg ml^−1^) for 16 h in the presence Torin 1 (50 nM) or vehicle control. Intracellular metabolites, isolated using methanol extraction of two technical replicates, were analyzed using the Ultimate 3000 UHPLC (Dionex) coupled to Q Exactive Plus-Mass spectrometer (QE-MS, Thermo Fisher Scientific) for metabolite profiling. Detailed methods were previously described^68^, except that mobile phase A was replaced with water containing 5 mM ammonium acetate (pH 6.8). Differentially expressed metabolites were identified by Limma (Bioconductor) and the Benjamini-Hochberg method was used to estimate the false discover rate (FDR). MetaboAnalyst was used to analyze range-scale data and provide KEGG pathway analysis of significantly altered metabolic pathways (log_2_ = 0.5) (www.metaboanalyst.ca/)^49^.

### Gene expression analysis

For mTOR deletion efficiency in T_reg_ cells, quantitative real-time PCR analysis was performed using *Mtor* Taqman probes (Thermo Fisher Scientific, Cat #4351372). For detection of *Il4* and *Il21*, CD4^+^Foxp3-YFP^–^CD44^hi^CXCR5^–^PD-1^−^ non-T_FH_ cells or CD4^+^Foxp3-YFP^–^CD44^hi^CXCR5^+^PD-1^+^ T_FH_ cells were stimulated for 4 h using plate bound anti-CD3 (5 μg ml^−1^) and anti-CD28 (5 μg ml^−1^) antibodies. Quantitative real-time PCR analysis was performed using SyBR Green Real-Time PCR Master Mix (Thermo Fisher Scientific) and primers for *Il4* (Forward 5’-GGTCTCAACCCCCAGCTAGT-3’; Reverse 5’-GCCGATGATCTCTCTCAAGTGAT-3’) and *Il21* (Forward 5’-GGACCCTTGTCTGTCTGGTAG-3’; Reverse 5’-TGTGGAGCTGATAGAAGTTCAGG-3’). For microarray analysis, RNA samples from unstimulated cT_reg_ cells or cT_reg_ cells activated in the presence of vehicle, Torin 1, or PP242 for 20 h as indicated above were analyzed with the GeneChip Mouse Gene 2.0 ST Array (Thermo Fisher Scientific). Differentially expressed transcripts in biological triplicate samples were identified by ANOVA (Partek Genomics Suite version 6.5), and the Benjamini-Hochberg method was used to estimate the FDR.

GSEA of hallmark pathways in resting vs. activated T_reg_ cells from these microarray samples or published datasets (GSE55753^32^ or GSE61077^7^) was performed as previously described^30^. IRF4 targets were identified from ChIP-seq data^22^ deposited in GSE98263 and compared against genes that were differentially expressed in activated cT_reg_ cells treated with or without mTOR inhibitors as above. IRF4 target genes that were differentially expressed in activated cT_reg_ cells treated with mTOR inhibitors were subjected to functional enrichment analysis of metabolism-related pathways, where significance was determined using the Fisher exact test and Benjamini-Hochberg method (FDR < 0.05).

### Statistics

The results in graphs represent the mean ± s.e.m., with the numbers of mice per group and number of experimental replicates indicated in each figure legend. The *P*-values were calculated with unpaired, two-tailed Student’s *t*-test assuming equal variance (GraphPad Prism software), where *P < 0.05; **P < 0.01; ***P < 0.001. No specific randomization methods were used in these studies. Investigators were not blinded to samples except where indicated for histological and immunohistochemical analysis.

### Data availability

Microarray data that support the findings of this study have been deposited in the Gene Expression Omnibus with the primary accession code GSE104130. Other data are available from the corresponding author upon request.

## Electronic supplementary material

Supplementary Information
