# Supplementary material for: mTOR coordinates transcriptional programs and mitochondrial metabolism of activated Treg subsets to protect tissue homeostasis
Source: Nat Commun. 2018 May 29;9:2095. doi: 10.1038/s41467-018-04392-5 (PMC5974344; doi:10.1038/s41467-018-04392-5)
Supplement: Supplementary file 1 — Supplementary Information [file 41467_2018_4392_MOESM1_ESM.pdf]

SUPPLEMENTARY INFORMATION

mTOR coordinates transcriptional programs and mitochondrial metabolism of activated T_{reg} subsets to protect tissue homeostasis

Chapman *et al.*

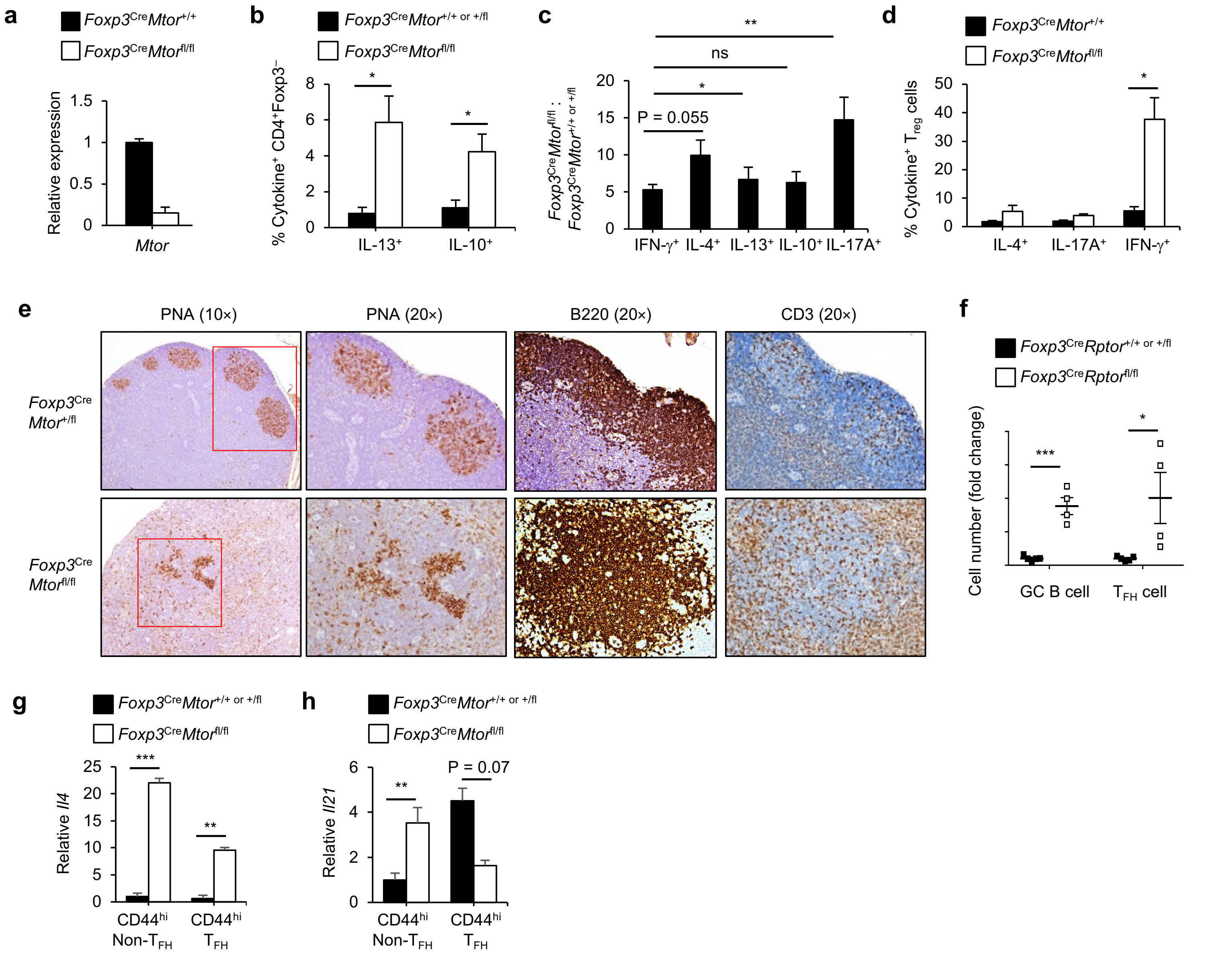

Supplementary Fig. 1. *Foxp3*^{Cre}-mediated deletion of *Mtor* in T_{reg} cells leads to altered cytokine production and GC responses. **a** Deletion efficiency of *Mtor* in T_{reg} cells as assessed by qPCR. **b** Quantification of IL-13- and IL-10-producing $CD4^+Foxp3^–$ T cells in *Foxp3*^{Cre}*Mtor*$^{+/+\text{ or }+/fl}$ or *Foxp3*^{Cre}*Mtor*$^{fl/fl}$ mice. **c** Quantification of the fold change of IFN-γ-, IL-4-, IL-13-, IL-10-, and IL-17A-producing $CD4^+Foxp3^–$ T cells in the spleen of *Foxp3*^{Cre}*Mtor*$^{fl/fl}$ mice compared to *Foxp3*^{Cre}*Mtor*$^{+/+\text{ or }+/fl}$ mice. **d** Quantification of IL-4-, IL-17A-, and IFN-γ-producing $CD4^+Foxp3^+$ T_{reg} cells in *Foxp3*^{Cre}*Mtor*$^{+/+\text{ or }+/fl}$ and *Foxp3*^{Cre}*Mtor*$^{fl/fl}$ mice. **e** Representative immunohistochemistry of PNA^+ cells, $B220^+$ B cells, and $CD3^+$ T cells in the mesenteric lymph nodes of *Foxp3*^{Cre}*Mtor*$^{+/+\text{ or }+/fl}$ and *Foxp3*^{Cre}*Mtor*$^{fl/fl}$ mice. The boxed regions in PNA (10×) are shown at higher power in PNA (20×). **f** Quantification of the fold change of GC B cells and T_{FH} cells in the spleen of *Foxp3*^{Cre}*Rptor*$^{+/+\text{ or }+/fl}$ and *Foxp3*^{Cre}*Rptor*$^{fl/fl}$ mice. **g, h** Analysis of *Il4* (**g**) and *Il21* (**h**) expression in $CD4^+$Foxp3-$YFP^–CD44^{hi}CXCR5^–$PD-$1^–$ non-T_{FH} cells and $CD4^+$Foxp3-$YFP^–CD44^{hi}CXCR5^+$PD-1^+ T_{FH} cells isolated from the spleen of *Foxp3*^{Cre}*Mtor*$^{+/+\text{ or }+/fl}$ or *Foxp3*^{Cre}*Mtor*$^{fl/fl}$ mice. Error bars show mean ± s.e.m. **P* < 0.05; ***P* < 0.01; ****P* < 0.001; ns, not significant; unpaired, two-tailed Student's *t*-test. Data are representative of three biological replicates (e) or are quantified from four (**a**), five (**b, c**, IL-10^+ cells), or six or eight (**b, c,** IL-13^+ cells in *Foxp3*^{Cre}*Mtor*$^{+/+\text{ or }+/fl}$ or *Foxp3*^{Cre}*Mtor*$^{fl/fl}$ mice, respectively), five (**d, f**), or three (**g, h**) biological replicates, compiled from two (**a, f**), five (**b**, IL-10^+ cells; **d**), six (**b**, IL-13^+ cells), twelve (**c**, IL-$17A^+$ cells), fourteen (**c**, IFN-$γ^+$ and IL-4^+ cells), or one (**g, h**) independent experiments.

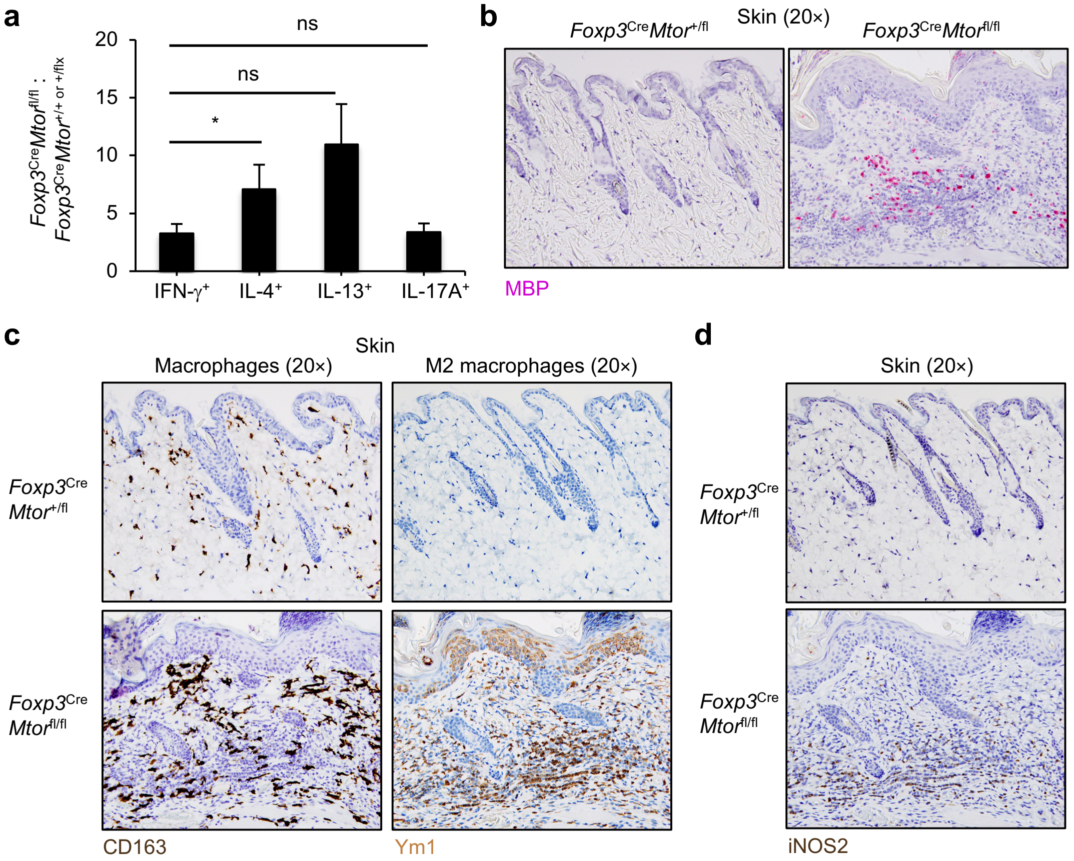

Supplementary Fig. 2. T_H2 responses are elevated in the lung and skin of *Foxp3*^{Cre}*Mtor*$^{fl/fl}$ mice**.**
a Quantification of the fold change of IFN-γ-, IL-4-, IL-13-, and IL-17A-producing $CD4^+Foxp3^-$ T cells in the lung of *Foxp3*^{Cre}*Mtor*$^{fl/fl}$ mice compared to *Foxp3*^{Cre}*Mtor*$^{+/+ \text{ or } +/fl}$ mice. **b** Representative images of major basic protein (MBP) staining for eosinophils in the skin of *Foxp3*^{Cre}*Mtor*$^{+/fl}$ and *Foxp3*^{Cre}*Mtor*$^{fl/fl}$ mice. **c** Representative images for M2 macrophages in the skin of *Foxp3*^{Cre}*Mtor*$^{+/fl}$ or *Foxp3*^{Cre}*Mtor*$^{+/fl}$ mice by CD163 and Ym1 staining. **d** Representative images for neutrophils, as indicated by inducible nitric oxide synthease 2 (iNOS2) staining, in the skin of *Foxp3*^{Cre}*Mtor*$^{+/fl}$ and *Foxp3*^{Cre}*Mtor*$^{fl/fl}$ mice. Error bars show mean ± s.e.m. *$P < 0.05$; ns, not significant; unpaired, two-tailed Student's *t*-test. Data are representative of three biological replicates per group (**b**–**d**) or are quantified from eight (**a**) biological replicates per group, compiled from four independent experiments (**a**).

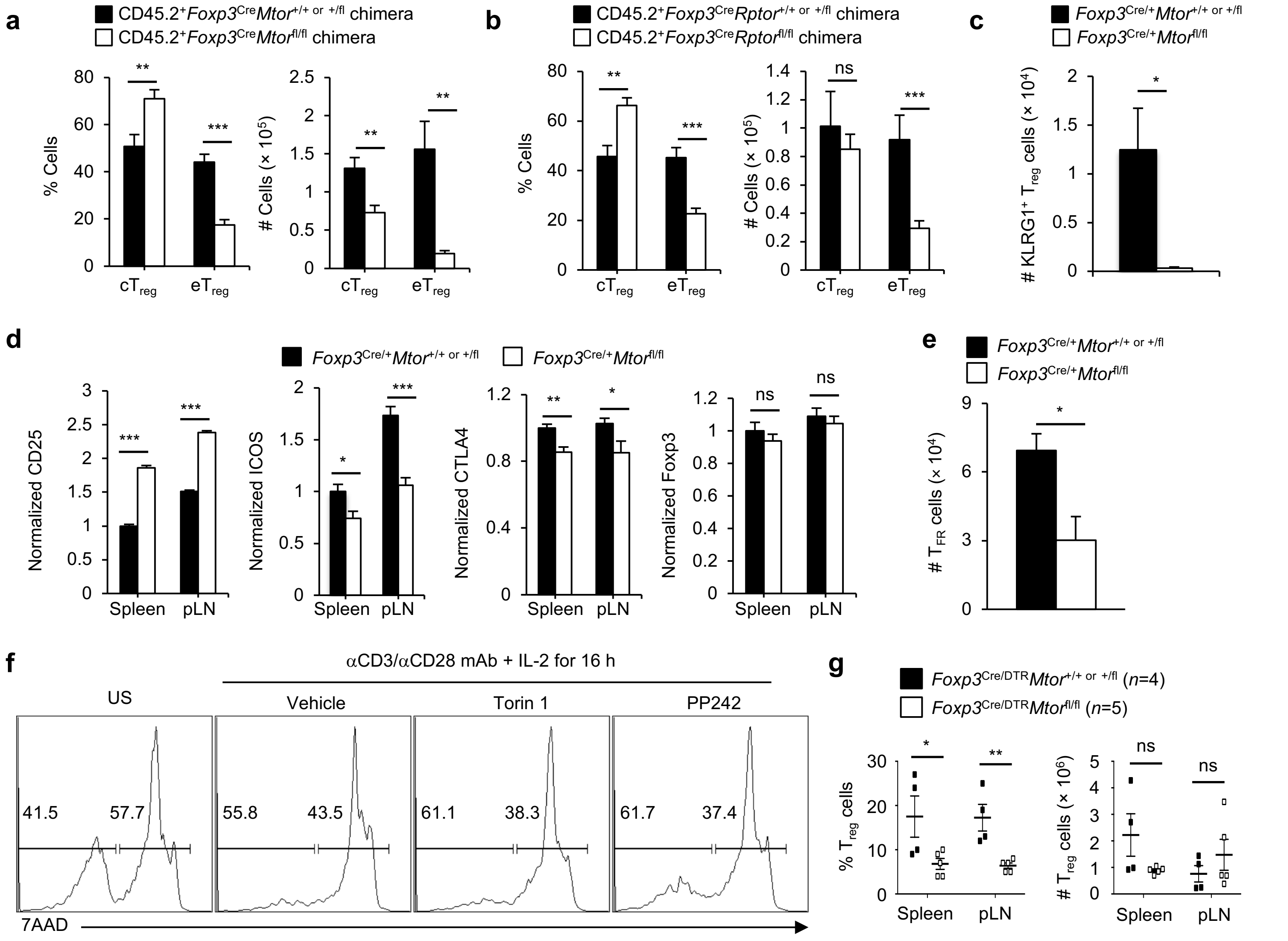

Supplementary Fig. 3. mTOR supports activated T_{reg} cell expansion through a survival-independent mechanism. **a** Quantification of frequencies and cell numbers of mTOR-deficient $CD4^+$Foxp3-$YFP^+CD44^{lo}CD62L^{hi}$ cT_{reg} cells and $CD4^+$Foxp3-$YFP^+CD44^{hi}CD62L^{lo}$ eT_{reg} cells in mixed bone marrow chimeras. **b** Quantification of Raptor-deficient $CD4^+$Foxp3-$YFP^+CD44^{lo}CD62L^{hi}$ cT_{reg} cells and $CD4^+$Foxp3-$YFP^+CD44^{hi}CD62L^{lo}$ eT_{reg} cells in mixed bone marrow chimeras. **c** Quantification of the number of $KLRG1^+$ T_{reg} cells in the spleen of *Foxp3*$^{Cre/+}$*Mtor*$^{+/+ \text{ or } +/fl}$ and *Foxp3*$^{Cre/+}$*Mtor*$^{fl/fl}$ mosaic mice. **d** Quantification of CD25, ICOS, CTLA4, and Foxp3 expression in T_{reg} cells in the spleen and peripheral lymph nodes (pLN) from *Foxp3*$^{Cre/+}$*Mtor*$^{+/+ \text{ or } +/fl}$ and *Foxp3*$^{Cre/+}$*Mtor*$^{fl/fl}$ mosaic mice. **e** Quantification of the number of T_{FR} cells ($CD4^+$Foxp3-$YFP^+CXCR5^+PD\text{-}1^+$ T cells) in *Foxp3*$^{Cre/+}$*Mtor*$^{+/+ \text{ or } +/fl}$ and *Foxp3*$^{Cre/+}$*Mtor*$^{fl/fl}$ mosaic mice. **f** cT_{reg} cells were activated in vitro for 16 h with anti-CD3 and anti-CD28 antibodies in the presence of IL-2. 7AAD staining was used to distinguish between live ($7AAD^-$) and dead ($7AAD^+$) cells. **g** Quantification of the frequencies and numbers of Foxp3-YFP^+ T_{reg} cells in the spleen and pLN of *Foxp3*$^{Cre/DTR}$*Mtor*$^{+/+ \text{ or } +/fl}$ or *Foxp3*$^{Cre/DTR}$*Mtor*$^{fl/fl}$ mosaic mice following diphtheria toxin (DT) treatment. Error bars show mean ± s.e.m. * $P < 0.05$; ** $P < 0.01$; *** $P < 0.001$; ns, not significant; unpaired, two-tailed Student's *t*-test. Data are representative of two independent experiments (**f**) or are quantified from twelve or thirteen (**a**, $CD45.2^+$*Foxp3*^{Cre}*Mtor*$^{+/+ \text{ or } +/fl}$ chimera and $CD45.2^+$*Foxp3*^{Cre}*Mtor*$^{fl/fl}$ chimera, respectively), five or seven (**b**, $CD45.2^+$*Foxp3*^{Cre}*Rptor*$^{+/+ \text{ or } +/fl}$ chimera and $CD45.2^+$*Foxp3*^{Cre}*Rptor*$^{fl/fl}$ chimera, respectively), eight (**c**; **d**, CD25, CTLA4, and Foxp3), nine (**d**, ICOS), six (**e**), or three or four (**g**, as indicated) biological replicates, compiled from seven (**a**), four (**b**; **c**; **d**, CD25, CTLA4, and Foxp3), five (**d**, ICOS), three (**e**), or two (**g**) independent experiments. Numbers indicate percentage of cells in gates.

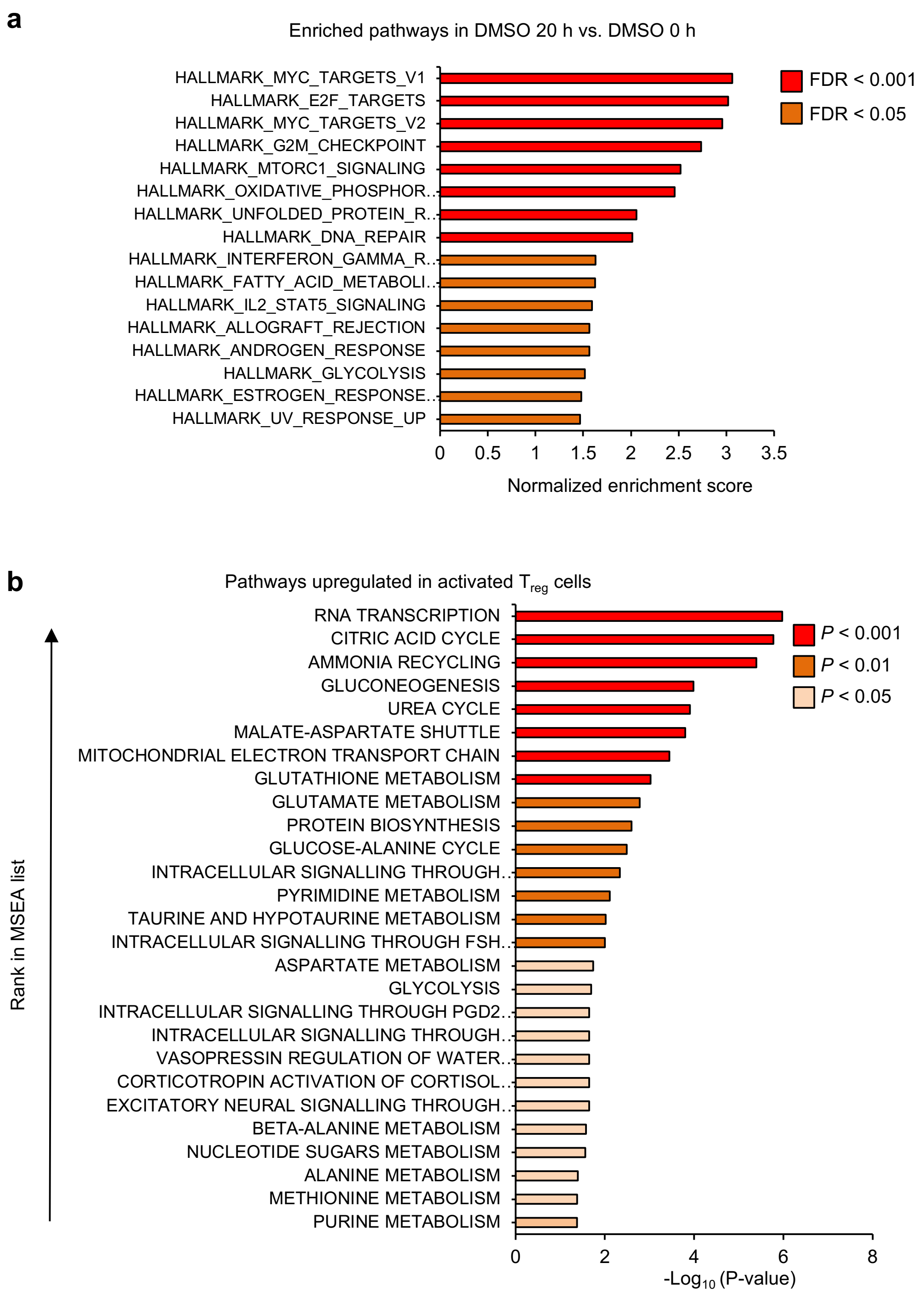

Supplementary Fig. 4. Characterization of mitochondrial metabolism in T_{reg} cells. **a** GSEA identified Hallmark pathways enriched in cT_{reg} cells activated for 20 h compared to unstimulated controls. **b** Metabolomics and metabolite set enriched analysis (MSEA) were used to identify various KEGG pathways upregulated in activated T_{reg} cells vs. unstimulated T_{reg} cells.

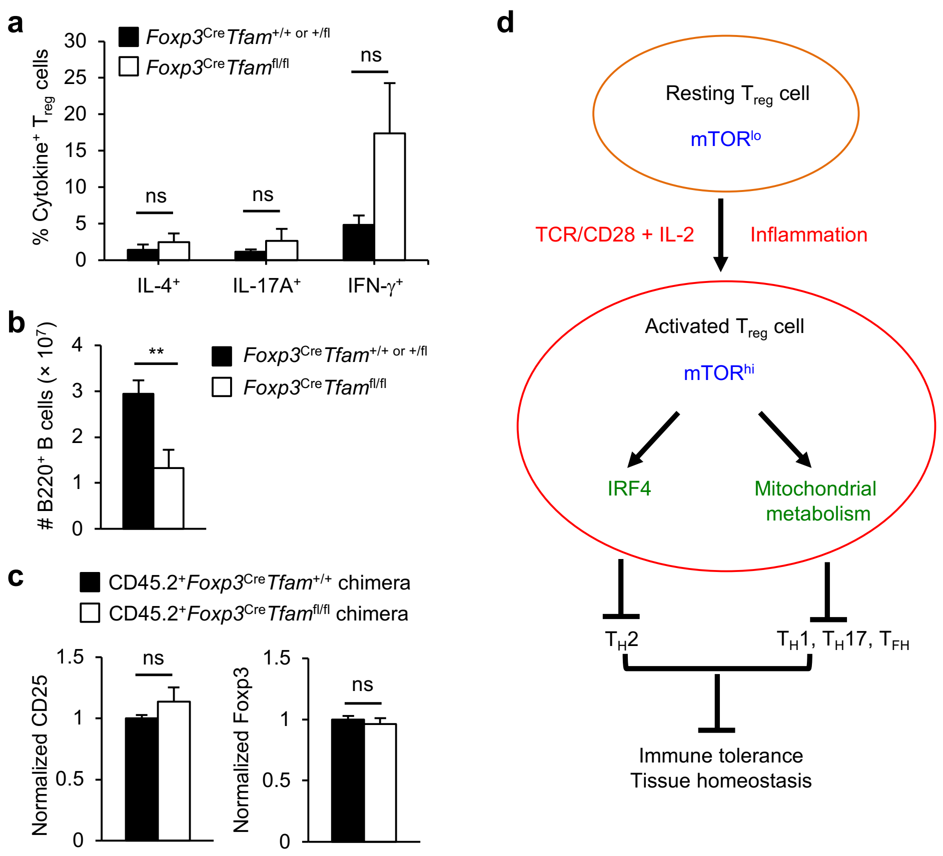

Supplementary Fig. 5. Characterization of T_{reg} and B cell populations in *Foxp3*^{Cre}*Tfam*$^{fl/fl}$ mice and model for mTOR-dependent coordination of IRF4 and mitochondrial metabolism in programming T_{reg} cell function. **a** Quantification of IL-4-, IL-17A-, and IFN-γ-producing $CD4^+Foxp3^+$ T_{reg} cells in *Foxp3*^{Cre}*Tfam*$^{+/+ \text{ or } +/fl}$ and *Foxp3*^{Cre}*Tfam*$^{fl/fl}$ mice. **b** Quantification of the numbers of $B220^+$ B cells in the spleen of *Foxp3*^{Cre}*Tfam*$^{+/+ \text{ or } +/fl}$ and *Foxp3*^{Cre}*Tfam*$^{fl/fl}$ mice. **c** Quantification of CD25 and Foxp3 expression in T_{reg} cells from mixed bone marrow chimeras. Error bars show mean ± s.e.m. ** $P < 0.01$; ns, not significant; unpaired, two-tailed Student's *t*-test. Data are quantified from six (**a**, $IL\text{-}4^+$ cells), nine (**a**, $IL\text{-}17A^+$ and IFN-γ^+ cells), ten (**b**), or nine or ten (**c**, $CD45.2^+$*Foxp3*^{Cre}*Tfam*$^{+/+}$ chimeras and $CD45.2^+$*Foxp3*^{Cre}*Tfam*$^{+/fl}$ chimeras, respectively) biological replicates, compiled from six (**a**, $IL\text{-}4^+$ cells), nine (**a**, $IL\text{-}17A^+$ and IFN-γ^+ cells), seven (**b**), or four (**c**) independent experiments. **d** In the periphery, TCR, co-stimulatory, IL-2, and inflammatory signals activate mTOR function in resting T_{reg} cells to promote IRF4 expression and mitochondrial metabolism. These pathways coordinately control T_{reg} cell activation and homeostasis. In turn, activated T_{reg} cells maintain peripheral T cell tolerance and tissue homeostasis.

Supplementary Table 1. GSEA of cT_{reg} activated with mTOR inhibitors vs. DMSO control for 20 h

	Torin 1 20 h vs. DMSO 20 h			PP242 20 h vs. DMSO 20 h		
NAME	NES	NOM p-val	FDR q-val	NES	NOM p-val	FDR q-val
HALLMARK_E2F_TARGETS	-3.39	< 0.001	< 0.001	-3.31	< 0.001	< 0.001
HALLMARK_G2M_CHECKPOINT	-3.27	< 0.001	< 0.001	-3.04	< 0.001	< 0.001
HALLMARK_MYC_TARGETS_V1	-3.16	< 0.001	< 0.001	-3.08	< 0.001	< 0.001
HALLMARK_MTORC1_SIGNALING	-2.89	< 0.001	< 0.001	-2.78	< 0.001	< 0.001
HALLMARK_OXIDATIVE_PHOSPHORYLATION	-2.79	< 0.001	< 0.001	-2.70	< 0.001	< 0.001
HALLMARK_MYC_TARGETS_V2	-2.66	< 0.001	< 0.001	-2.87	< 0.001	< 0.001
HALLMARK_MITOTIC_SPINDLE	-2.21	< 0.001	< 0.001	-2.16	< 0.001	1.56E-04
HALLMARK_UNFOLDED_PROTEIN_RESPONSE	-2.20	< 0.001	< 0.001	-1.82	< 0.001	4.95E-04
HALLMARK_DNA_REPAIR	-2.12	< 0.001	< 0.001	-2.17	< 0.001	1.79E-04
HALLMARK_INTERFERON_GAMMA_RESPONSE	-1.95	< 0.001	1.32E-04	-1.63	< 0.001	6.90E-03
HALLMARK_FATTY_ACID_METABOLISM	-1.84	< 0.001	6.82E-04	-2.05	< 0.001	1.25E-04
HALLMARK_GLYCOLYSIS	-1.81	< 0.001	9.61E-04	-1.97	< 0.001	1.14E-04
HALLMARK_ESTROGEN_RESPONSE_LATE	-1.79	< 0.001	1.13E-03	-1.75	< 0.001	1.29E-03
HALLMARK_ADIPOGENESIS	-1.75	< 0.001	1.88E-03	-1.64	< 0.001	6.13E-03
HALLMARK_UV_RESPONSE_UP	-1.63	< 0.001	7.60E-03	-1.71	< 0.001	2.24E-03
HALLMARK_CHOLESTEROL_HOMEOSTASIS	-1.62	1.32E-02	8.21E-03	-2.06	< 0.001	1.39E-04
HALLMARK_SPERMATOGENESIS	-1.58	< 0.001	1.28E-02	-1.62	< 0.001	6.74E-03
HALLMARK_XENOBIOTIC_METABOLISM	-1.53	< 0.001	1.75E-02	-1.48	2.99E-03	2.18E-02
HALLMARK_ALLOGRAFT_REJECTION	-1.51	5.21E-03	2.05E-02	-1.58	< 0.001	9.49E-03
HALLMARK_PI3K_AKT_MTOR_SIGNALING	-1.41	2.40E-02	4.64E-02	-1.40	3.48E-02	4.19E-02
